# Hydroxychloroquine-induced Retinal Toxicity

**DOI:** 10.3389/fphar.2023.1196783

**Published:** 2023-05-30

**Authors:** Imran H. Yusuf, Peter Charbel Issa, Seong Joon Ahn

**Affiliations:** ^1^ Oxford Eye Hospital and Nuffield Department of Clinical Neurosciences, John Radcliffe Hospital, University of Oxford, Oxford, United Kingdom; ^2^ Department of Ophthalmology, Hanyang University Hospital, Hanyang University College of Medicine, Seoul, South Korea

**Keywords:** hydroxychloroquine, optical coherence tomography, retinal imaging, screening, toxicity, retinopathy, hydroxychloroquine retinopathy

## Abstract

Long-term use of hydroxychloroquine can cause retinopathy, which may result in severe and progressive visual loss. In the past decade, hydroxychloroquine use has markedly increased and modern retinal imaging techniques have enabled the detection of early, pre-symptomatic disease. As a consequence, the prevalence of retinal toxicity in long-term hydroxychloroquine users is known to be higher than was previously estimated. The pathophysiology of the retinopathy is incompletely characterised, although significant advances have been made in understanding the disease from clinical imaging studies. Hydroxychloroquine retinopathy elicits sufficient public health concern to justify the implementation of retinopathy screening programs for patients at risk. Here, we describe the historical background of hydroxychloroquine retinopathy and summarize its current understanding. We review the utility and limitations of each of the mainstream diagnostic tests used to detect hydroxychloroquine retinopathy. The key considerations towards a consensus on the definition of hydroxychloroquine retinopathy are outlined in the context of what is known of the natural history of the disease. We compare the current screening recommendations for hydroxychloroquine retinopathy, identifying where additional evidence is required, and the management of proven cases of toxicity. Finally, we highlight the areas for further investigation, which may further reduce the risk of visual loss in hydroxychloroquine users.

## 1 Introduction: Historical and medical background

Hydroxychloroquine retinopathy is a drug-induced, or toxic, retinopathy first described in the 1960s in users of hydroxychloroquine (Scherbel et al., 1965; Shearer and Dubois, 1967). The use of hydroxychloroquine has increased significantly over the past 20–30 years (Yates et al., 2018), in part due to a more aggressive treatment approach to chronic inflammatory disorders, its relatively low cost and safety compared to novel disease-modifying agents. Furthermore, hydroxychloroquine use has been strongly supported by the demonstration of a survival benefit in patients with systemic lupus erythematosus (Alarcon et al., 2007).

Hydroxychloroquine retinopathy was previously considered rare since it was typically diagnosed at an advanced stage with symptoms of central visual loss and visible fundoscopic changes, such as “bull’s eye” maculopathy, characterized by loss of retinal pigment epithelium. However, the advent of optical coherence tomography (OCT) imaging has enabled the detection of pre-symptomatic retinal damage, before fundus changes are visible. This has fundamentally changed the approach to disease detection, dosing recommendations and screening procedures. Consequently, it is now possible to detect early retinopathy in a large number of individuals at risk of potentially avoidable visual loss, enabling treatment cessation before the disease becomes progressive. Screening is now recommended in order to reduce the risk of visual loss from toxic retinopathy in hydroxychloroquine users through the detection of early changes typical of the disease. This article highlights some earlier data; however, with the focus on hydroxychloroquine retinopathy screening/management in the OCT era.

### 1.1 Discovery and initial usage of chloroquine

Hydroxychloroquine (Plaquenil^®^) and its parent drug, chloroquine, are synthetic drugs originally developed to treat malaria. The first anti-malarial agent developed was quinacrine (Atabrine^®^), which was used to treat malaria in the South Pacific during the Second World War (Ellerbrook and Lippincott, 1945). However, the toxic effects of quinacrine, which involved fluorescent pigmentation of tissues, agranulocytosis, retinal toxicity (Browning, 2004), and psychosis (Lutterloh and Shallenberger, 1946; Sheppeck and Wexberg, 1946), led to the pursuit of less toxic quinacrine analogs through modification of the alpha side-chain (Bloom et al., 1945). Chloroquine was discovered in 1934 through the chemical modification of quinacrine (Schroeder and Gerber, 2014), and tested in the field by the United States (US) army in 1943 (Goldsmith, 1946), where it was shown to be more efficacious than quinacrine and quinine (Pullman et al., 1948).

Chloroquine has been used as a first-line treatment for malaria for decades. During this period, the use of chloroquine and other anti-malarial drugs has become more widespread in the treatment of systemic lupus erythematosus (SLE) and rheumatoid arthritis (RA). Although chloroquine is typically used for short courses for antimalarial treatment, higher doses and a longer duration of therapy are required for the treatment of rheumatic diseases. In addition to the systemic adverse effects of chloroquine, dose-dependent adverse effects on the visual system were documented in the late 1940s (Dekking, 1949). Chloroquine retinopathy was identified as a clinical entity in 1959 (Hobbs et al., 1959), occurring as early as 4 years after initiating therapy (Ormrod, 1962). Thus, due to the observed toxicity of chloroquine, hydroxychloroquine was developed through the chemical addition of an hydroxyl group (Hoekenga, 1955).

### 1.2 Transition to hydroxychloroquine

Hydroxychloroquine was established as a systemically less toxic alternative to chloroquine in the 1940–50s. Over the ensuing 70 years, the anti-inflammatory, anti-thrombotic, and anti-neoplastic effects of hydroxychloroquine were discovered. Hydroxychloroquine has been repurposed in the long-term management of numerous chronic inflammatory disorders and is considered more cost-effective than novel alternative anti-inflammatory agents, particularly biological agents (Eriksson et al., 2015).

The favorable safety profile of hydroxychloroquine in comparison to other systemic immunomodulatory drugs, its relatively low cost (Erkan et al., 2002), increasing evidence of its efficacy as a disease-modifying agent, and a proven survival benefit in SLE (Alarcon et al., 2007) has led to a significant increase in the number of hydroxychloroquine prescriptions in the developed world. For example, in the United Kingdom (UK), it is estimated that there are approximately 11,000 new initiators of hydroxychloroquine annually; 51.7% of newly diagnosed patients with RA and 67% of those with newly diagnosed SLE are started on the drug (Yates et al., 2018). Furthermore, prescribing data suggest that around 100,000 individuals take hydroxychloroquine in the United Kingdom, and the number of prescriptions has increased significantly from 2010 to 2016 (Yates et al., 2018). These data suggest that the number of individuals exposed to hydroxychloroquine and, therefore, at risk of potential side effects is increasing.

The first case of hydroxychloroquine-related retinopathy was identified in 1965 in a patient taking 1 g of hydroxychloroquine daily (Scherbel et al., 1965) with additional reports thereafter (Shearer and Dubois, 1967). However, in contrast to chloroquine, symptomatic retinal toxicity was detected rarely in hydroxychloroquine users over a similar period of treatment (Rynes et al., 1979). The improved safety profile of hydroxychloroquine led to the almost exclusive use of hydroxychloroquine above that of other antimalarials in the treatment of rheumatologic disease in the US in the late 1970s (Rynes et al., 1979). By the mid-1990s, only 20 cases of hydroxychloroquine retinopathy had been reported in the literature—however, the diagnosis was usually only made in symptomatic patients with severe disease that could be detected on fundoscopy (Rynes and Bernstein, 1993). Later, the emergence of modern retinal imaging techniques has enabled the detection of more subtle changes indicative of early toxic retinopathy before the development of symptoms. Using such techniques, the prevalence of retinopathy was estimated to be approximately 7.5% in patients taking the drug for over 5 years and 20%–50% after 20 years (Melles and Marmor, 2014). Given the large numbers of patients taking long-term hydroxychloroquine and the availability of modern retinal imaging technology, hydroxychloroquine retinopathy has become a significant public health concern, justifying the monitoring or screening of at-risk groups (Marmor et al., 2016; Yusuf et al., 2021b).

### 1.3 Chemistry and systemic/ocular pharmacokinetics

**FIGURE 1:**
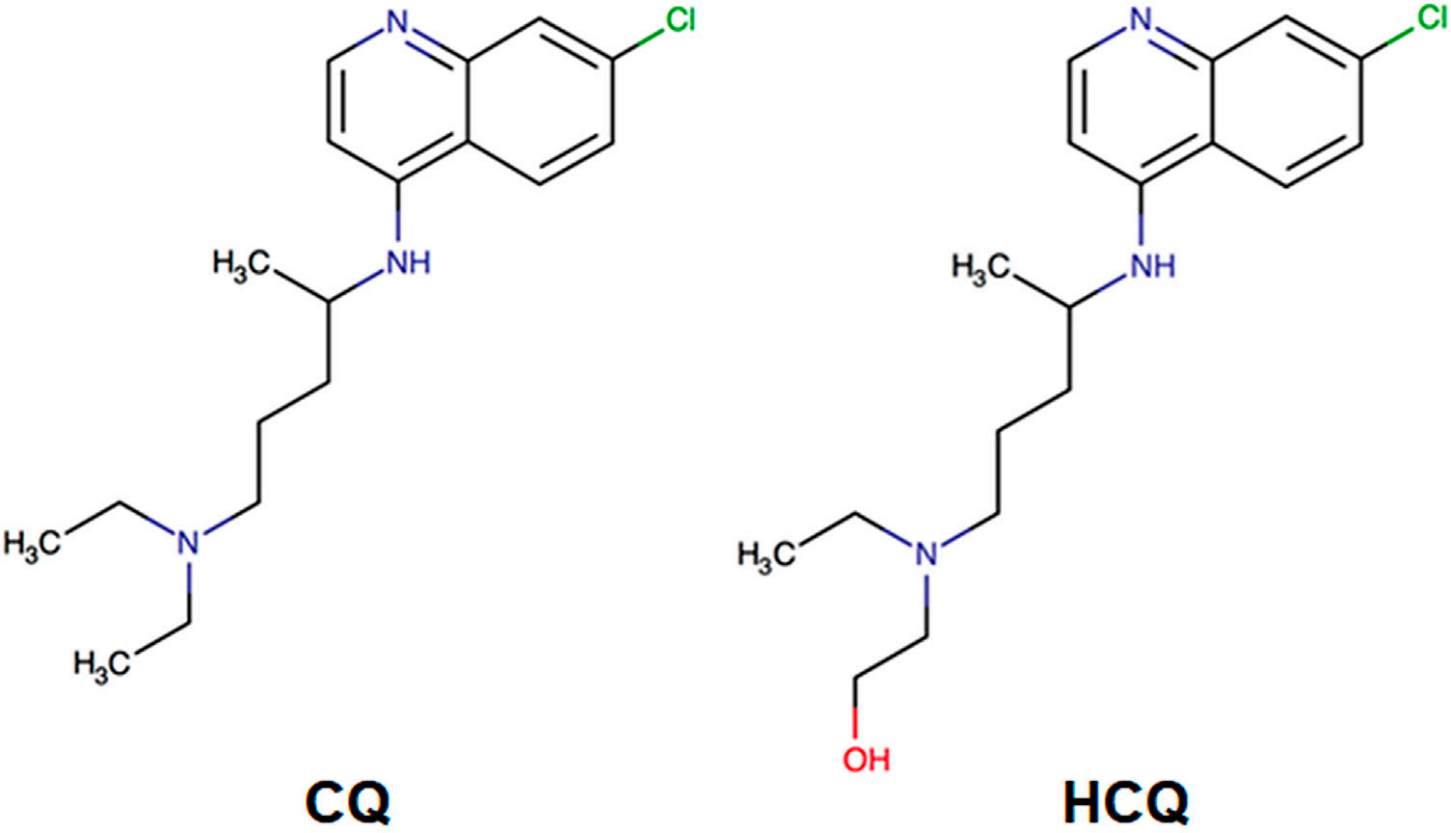
The molecular structure of chloroquine (left) and hydroxychloroquine (right).

Hydroxychloroquine is an organic 4-aminoquinoline compound produced from a quinoline (a heterocyclic aromatic compound formed from the cyclization of o-substituted benzenes) ring with an alpha side-chain containing a hydroxyl group at the terminal end of the chain (Figure 1). Quinolines are generally non-toxic to humans at low doses. Chemical modification of the quinoline ring scaffold has produced numerous classes of therapeutic agents, such as anti-inflammatory, antibiotics, and anti-neoplastic agents (Chu et al., 2019). The hydroxyl group differentiates hydroxychloroquine from chloroquine.

Hydroxychloroquine is administered orally and is rapidly absorbed by the gastrointestinal tract. Mean gastrointestinal absorption of hydroxychloroquine was 74% (SD, ±13%) following oral administration in one study (Tett et al., 1989). Hydroxychloroquine is transported in the plasma with 45% of the drug bound to albumin and other serum proteins and has a volume of distribution of 44,000 L from plasma, indicating a very high distribution of hydroxychloroquine into tissues (Rainsford et al., 2015). The half-life of the drug in plasma has been reported to be approximately 40 days, ranging from 32 to 50 days (Tett et al., 1989; Titus, 1989; Rand et al., 2008). Hydroxychloroquine is metabolized by dealkylation in the liver (Ducharme and Farinotti, 1996). Due to its high sequestration in tissues, hemodialysis is not effective in removing the drug following overdose. Following once-daily administration, a steady-state concentration was achieved in the plasma after approximately 6 months in one study (Tett et al., 1989). Renal excretion accounts for 22%–60% of the total drug and metabolites (Berliner et al., 1948; Mackenzie, 1983a; Titus, 1989; Miller et al., 1991; Albert et al., 1998; Ono et al., 2003; Kalia and Dutz, 2007). Chronic renal failure is considered one of the major risk factors for toxicity, and unusual cases of early or rapid toxicity can be noted among patients within this group.

The ocular distribution of systemically administered hydroxychloroquine has been studied in rabbit models (Wainer et al., 1994). Hydroxychloroquine is detectable in both pigmented and non-pigmented structures within the eye. In pigmented structures, such as the ciliary body, choroid, and retinal pigment epithelium (RPE), hydroxychloroquine is bound to melanin (Wainer et al., 1994). However, hydroxychloroquine accumulation within the retina has not been studied in detail. Compared to chloroquine, hydroxychloroquine showed lower tissue accumulation in melanin-rich tissues; however, it is unclear whether this finding alone explains the lower risk of retinal toxicity from hydroxychloroquine (Schroeder and Gerber, 2014).

### 1.4 Medical indications and uses

Antimalarials have a complex mode of action through which anti-inflammatory, immunomodulatory, anti-infective, anti-thrombotic, and other metabolic effects are exerted. This has led to its widespread use in rheumatology, dermatology, pediatrics, oncology, and infectious diseases. Table 1 summarizes the current treatment indications for hydroxychloroquine.

**TABLE 1:** Indications for hydroxychloroquine use.

**Infectious disease**
For the prophylaxis and treatment of acute malaria due to *P. vivax*, *P. ovale*, *P. malariae* and susceptible strains of P. falciparum (approved*)
Amoebiasis (extra-intestinal)
Q fever (*Coxiella Burnetti*)
**Rheumatologic disease**
Rheumatoid arthritis (approved^a^)
Systemic lupus erythematosus (approved^a^)
Sjogrens syndrome
Mixed connective tissue disease
Anti-phospholipid syndrome
Sarcoidosis
**Dermatological disease**
Discoid lupus erythematosus (DLE)
Photosensitive dermatoses (polymorphic light eruption, porphyria cutanea tarda, etc.)
Morphoea
Cutaneous sarcoidosis
Dermatomyositis
Granuloma annulare
Lichen planus
Urticaria
Alopecia areata
**Pulmonary disease**
Sarcoidosis
Interstitial lung disease (including pediatric)
**Hematology/oncology** (adjuvant use)
Non-small cell lung cancer (adjuvant)
Immune thrombocytopenic purpura
**Others**
Chronic graft *versus* host disease

^a^ Approved by the U.S., Food and Drug Administration (FDA).

In SLE, hydroxychloroquine monotherapy is often highly effective (Hanaoka et al., 2019), and given that the drug is systemically well tolerated, patients often continue the drug for many years (Wang et al., 1999). Hydroxychloroquine has proven efficacious in reducing the number of disease flares, preventing end-organ damage, permitting a reduction in systemic corticosteroid dose, and exerting an anti-thrombotic effect through a reduction in serum anti-phospholipid antibodies (Alarcon et al., 2007; Pons-Estel et al., 2009; Willis et al., 2012; Mok et al., 2016; Ponticelli and Moroni, 2017; Hsu et al., 2018). Given the reduction in mortality associated with hydroxychloroquine in SLE, the European League Against Rheumatism (EULAR) consensus in 2019 recommended that all patients with SLE should be treated with hydroxychloroquine at a dose of less than 5 mg/kg of real body weight (Fanouriakis et al., 2019).

In RA, hydroxychloroquine is often used in combination with other conventional, synthetic, disease-modifying anti-rheumatic drugs (csDMARDs). A recent systematic review evaluating the effect of hydroxychloroquine in combination with csDMARDs found that the inclusion of hydroxychloroquine is likely to result in clinical improvement in joint symptoms, efficacy on both clinical and biochemical outcome measures, and beneficial effects on the coexistent heart or kidney disease and serum lipids in patients with RA (Gaujoux-Viala et al., 2010; Restrepo et al., 2017; Hung et al., 2018; Wu et al., 2018; Rempenault et al., 2019; Schapink et al., 2019). Collectively, these data suggest a useful role of hydroxychloroquine in the long-term management of patients with RA.

In dermatology, systemic hydroxychloroquine administration is used in the management of photosensitive dermatoses (such as discoid lupus erythematosus and polymorphic light eruption), systemic inflammatory disorders with cutaneous manifestations (such as dermatomyositis and cutaneous sarcoidosis), and others such as morphea and granuloma annulare. Dosing in these contexts may be intermittent, as the medication may be discontinued during the winter months when light exposure may be of shorter duration at certain latitudes.

The systemic safety of hydroxychloroquine has encouraged its use in children with diseases such as SLE, dermatomyositis, and porphyria cutanea, as hydroxychloroquine is used in the treatment of these disorders in adults. Moreover, hydroxychloroquine has been used in pediatric patients with chronic inflammatory disorders, such as immune thrombocytopenic purpura and interstitial lung disease (Braun et al., 2015). However, it should be noted that hydroxychloroquine is unlicensed for use in children. Further, hydroxychloroquine is considered as safe in pregnancy, leading to expert opinion recommendations that the drug can be continued during pregnancy and lactation (Costedoat-Chalumeau et al., 2005; Abarientos et al., 2011).

Hydroxychloroquine, as an inhibitor of autophagy, may lead to a reduction in tumor size, as shown in a preclinical rodent model of neuroendocrine neoplasia (Nakano et al., 2020). Extremely high daily doses are required to achieve inhibition of autophagy (often 1 g or more per day) within clinical trial regimens. Clinical trials on the adjuvant use of hydroxychloroquine have been performed in the context of small cell lung cancer, non-small cell lung cancer, pancreatic carcinoma, prostate cancer, colorectal cancer, renal cell carcinoma, and others (Mahalingam et al., 2014; Rangwala et al., 2014). However, hydroxychloroquine is not yet approved for use as an adjuvant therapy for any form of cancer with many studies currently in phase 2 clinical trial.

## 2 Pathogenesis of hydroxychloroquine ocular toxicity

The pathogenesis of hydroxychloroquine retinopathy has not been fully elucidated. Consequently, there has not been any suggestion of a therapeutic opportunity to intervene in the pathogenesis of the disease, except to discontinue the medication. In this section, we discuss the mechanisms of therapeutic action and summarize evidence on the pathogenic mechanisms of retinal toxicity from imaging studies in human eyes and studies utilizing *in vitro* techniques and animal models.

### 2.1 Mechanisms of therapeutic action

The malarial parasite must degrade hemoglobin, which consists of a globin chain and heme unit, to acquire essential amino acids. During digestion, the parasite releases toxic and soluble heme molecules. The parasite converts heme to form hemozoin, a non-toxic molecule. The major action of chloroquine is to inhibit the formation of hemozoin from heme. Free heme then lyses membranes, leading to parasite death. Chloroquine and hydroxychloroquine are lysosomotropic agents that accumulate preferentially in the lysosomes of human cells. The lysosomotropic character of chloroquine or hydroxychloroquine may partially explain its antimalarial activity as the drug concentrates in the acidic vacuole of the malarial parasite.

Chloroquine and hydroxychloroquine have a complex mode of action that confers anti-inflammatory, immunomodulatory, anti-infective, antithrombotic, and other metabolic effects. These drugs affect membrane stability and alter transcriptional activity, leading to the inhibition of cytokine production and modulation of several co-stimulatory molecules (Schrezenmeier and Dorner, 2020). The anti-inflammatory or immunomodulatory effect may originate from a significant decrease in the production of cytokines, particularly pro-inflammatory factors including interleukin (IL)-6 and IL-1β (Ornstein and Sperber, 1996; Fujita et al., 2019).

### 2.2 Mechanisms of toxicity

The pathogenesis of hydroxychloroquine-induced retinal toxicity is poorly understood. As chloroquine and hydroxychloroquine are known inhibitors of autophagy, these drugs alkalinize lysosomes, thereby, inhibiting lysosomal enzymes and arresting autophagy—although these mechanisms may be dose-dependent. Some observations from *in vitro* studies are summarized below. Human RPE-derived ARPE-19 cells were shown to undergo lysosomal enlargement, demonstrate an increase in autophagy markers and cell death in response to chloroquine treatment (Yoon et al., 2010). However, the specific effects on photoreceptors were not assessable in this *in vitro* study. Dose-dependent inhibition of rod photoreceptor outer segment phagocytosis by chloroquine was shown in two alternative human and pig-derived RPE cell lines (Mannerström et al., 2001).

Hydroxychloroquine was found to be a specific inhibitor of OATP1A2 expressed in human RPE cells, which enables the cellular uptake of all-trans-retinol as part of the classic visual cycle (Xu et al., 2015). This finding is potentially important as it directly links hydroxychloroquine with a specific cellular function at the level of the RPE critical to retinal physiology. High experimental doses also produce acute effects on the cellular metabolism of the retina, but it is unclear whether the short-term effects of excessive doses simulate the slowly progressive damage from chronic exposure seen in human eyes with retinopathy. *In vitro* studies into the mechanisms of hydroxychloroquine toxicity are limited by the inability to simulate chronic drug exposure, and the lack of suitable photoreceptor cell lines.

The role of light in hydroxychloroquine retinopathy might be suggested by the modifying effect of light toxicity in a patient with long-term unilateral aphakia with more severe disease in the contralateral phakic eye (Mack et al., 2018). In this context, the absence of focused light on the retina appeared to reduce the toxic effect of hydroxychloroquine on the retina despite what could be assumed as equal drug accumulation in pigmented tissues in both eyes (Mack et al., 2018). Further research is required to determine a potential modifier role of light in hydroxychloroquine retinopathy.

#### 2.2.1 Role of melanin

Melanin is a term given to a particular group of pigmented substances that are widely present in the tissues of living organisms. Within the eye, melanin is present within the iris stroma, posterior iris pigment epithelium, ciliary body (which together form the anterior choroid), posterior choroid, and RPE. Melanin has been shown to function as an important antioxidant molecule within the RPE (Wang et al., 2006). Kristensen et al. demonstrated the binding of hydroxychloroquine to melanin *in vitro* (Kristensen et al., 1994). Observations from animal or *in vitro* studies led to two dominant theories on the role of melanin in hydroxychloroquine retinopathy: that hydroxychloroquine binds to melanin enabling drug accumulation and toxicity and/or that hydroxychloroquine binding to melanin inhibits its role as an antioxidant, or as a protective molecule against light-toxicity.

Hydroxychloroquine was found to accumulate in pigmented and non-pigmented tissues in New Zealand white rabbits administered high doses of daily oral hydroxychloroquine (Wainer et al., 1994). In pigmented tissues, hydroxychloroquine was found to be bound to melanin, although the binding partner in non-pigmented tissues was not identified (Wainer et al., 1994). Schroeder directly compared the adsorption of melanin to both chloroquine and hydroxychloroquine (Schroeder and Gerber, 2014) and found a 45% reduction in the affinity of melanin in hydroxychloroquine when compared to chloroquine. It was hypothesized that binding to RPE melanin might prolong the toxic effects and that the increased affinity of melanin for chloroquine underlies the increased retinal toxicity of the drug per mg.

Melanin has been shown to play a protective role within the cell through metal ion chelation (Kaczara et al., 2012) (in some cases, much more efficiently than traditionally used metal ion chelators such as ethylene diamine tetraacetic acid), and exerts antioxidant effects through the scavenging of pro-oxidant systems. A comparative study of lipid peroxidation between pigmented and albino rabbits revealed reduced lipid peroxidation in the RPE of pigmented rabbits compared to albino rabbits, which appeared to be related to the presence of melanin-containing granules, while other prominent anti-oxidant cellular systems did not differ between the two animal groups (superoxide dismutase, glutathione peroxidase) (Ostrovsky et al., 1987). A further protective role of melanin in the RPE is the ability to absorb visible radiation. In adult chimeric mice with varied retinal pigmentation, which had been exposed to constant light for 5 weeks, rod photoreceptor cell death in poorly pigmented retinas and retinal locations was observed, suggesting a role of melanin in the protection of the retina against light toxicity (Sanyal and Zeilmaker, 1988).

### 2.3 Experimental models

Only a few basic science studies have been conducted to explore the primary effects of hydroxychloroquine and chloroquine on the RPE. One study examined the effects of antimalarials on cultured RPE cells fed with photoreceptor outer segments, finding that hydroxychloroquine was a less potent agent at stimulating lipofuscin formation when compared to chloroquine, owing to a weaker effect on lysosomal pH (Sundelin and Terman, 2002). A further study evaluated the effect of antimalarials on ARPE-19 cell monolayers *in vitro*, finding a significant increase in RPE permeability despite an increase in the expression of several proteins present in RPE intercellular tight junctions (Korthagen et al., 2015), appearing to confirm an earlier conclusion made following vitreous fluorophotometry in some patients with symptomatic disease (Raines et al., 1989). Antimalarials are lipophilic, which enhances their ability to cross the blood-brain barrier, and presumably, the inner blood-retinal barrier formed by microvascular endothelial cells. However, these data suggest an additional effect on RPE permeability, and some compromise of the outer blood-retinal barrier. This may further expose the retina to increasing concentrations of hydroxychloroquine and/or other drugs and toxins (Elhusseiny et al., 2019; Sharma et al., 2019) and contribute to retinal toxicity independent of a direct toxic effect of hydroxychloroquine.

Chloroquine induces vacuole formation and cell death in cultured ARPE-19 cells (Yoon et al., 2010). Although the mechanism of cell death in this study was not fully determined, it was hypothesized that an accumulation of certain toxic ubiquitinated proteins and the arrest of autophagy may play a role (Yoon et al., 2010). As zinc ionophore clioquinol re-acidifies lysosomes and reverses the arrest in chloroquine-treated ARPE-19 cells (Seo et al., 2015), its therapeutic effect on the outer retina may provide further insight into the pathogenesis of hydroxychloroquine retinopathy and may have some potential in ameliorating chloroquine/hydroxychloroquine toxicity (Koh et al., 2019). Further validation of the mouse model of chloroquine retinopathy (see below) may provide a method for the evaluation of candidate therapeutics. Further studies are required to examine the retinal changes following long-term administration of hydroxychloroquine or chloroquine to determine whether the *in vitro* observations following short-term exposure holds following chronic exposure to these drugs *in vivo*. Dose-dependent effects are known to be important in the cellular responses to hydroxychloroquine and chloroquine concerning their effects on lysosomal function (Chandler et al., 2020).

Studies on the *in vivo* effects of hydroxychloroquine retinopathy are hampered by the lack of suitable animal models. Rhesus macaques administered intramuscular chloroquine for 4.5 years did not show changes on fundus examination, fluorescein angiography, or electroretinography, although post-mortem analysis revealed extensive changes affecting the ganglion cell layer, photoreceptors, choroid, and RPE (Rosenthal et al., 1978). However, the study was undertaken before the advent of multimodal retinal imaging. Repeated investigation may be hampered by a high experimental cost of long-term non-human primate studies. More acute retinal changes were observed in *C57BL6J* mice that were administered an intraperitoneal injection of chloroquine for 62 days (Gaynes et al., 2008). A repeated study using modern retinal imaging techniques would be of interest to determine whether longitudinal changes in this disease model reflect those observed in patients.

## 3 Clinical characteristics of hydroxychloroquine ocular toxicity

### 3.1 Non-retinal effects

Systemic adverse effects of hydroxychloroquine are summarized in Table 2. Hydroxychloroquine may also adversely affect the cornea and the ciliary body. Cornea verticillata, or vortex keratopathy, is commonly found in patients taking chloroquine (Easterbrook, 1993). This is characterized by intraepithelial deposits in the cornea, composed of unchanged antimalarial drug. Beginning after 2–3 weeks to a few months of drug use, it presents with diffuse punctate deposits or aggregated forms as a vortex pattern. Patients with these corneal changes typically have no visual complaints or discomfort (Raizman et al., 2017).

**TABLE 2:** Systemic and ocular side effects in the users of hydroxychloroquine and chloroquine.

**Systemic adverse effects**
**Hepatotoxicity:** acute hepatic injury with elevated liver enzymes, acute hepatic failure
**Cardiac:** cardiomyopathy (restrictive), ventricular hypertrophy, cardiac conduction disorders
**Neurological:** seizures (interaction with mefloquine), tremor, myopathy (vacuolar) with risk of ventilatory failure, tinnitus, vertigo
**Psychiatric:** emotional lability and psychosis
**Haematological:** Blood dyscrasias (agranulocytosis, anemia, thrombocytopenia)
**Metabolic:** hypoglycemia
**Cutaneous:** Severe cutaneous adverse reactions including acute, generalized exanthematous pustulosis, erythroderma, toxic epidermal necrolysis, Sweet’s syndrome
Alopecia, hair color changes, pustular psoriasis
Angioedema

**Ocular side-effects**
Vortex keratopathy (cornea verticillata)
Outer retinal toxicity (hydroxychloroquine retinopathy)
Ciliary body dysfunction (not validated)

This keratopathy is extremely rare in patients taking hydroxychloroquine. This may reflect a greater tendency of chloroquine to accumulate in some non-pigmented tissues, apparently independent of melanin. Ciliary body involvement, manifesting in accommodative dysfunction, may occur after administration of chloroquine (Rubin and Thomas, 1970), whereas these adverse effects have not been reported in patients receiving hydroxychloroquine. These non-retinal effects are known to be generally reversible, in contrast to toxic retinopathy.

### 3.2 Retinal changes

Hydroxychloroquine retinopathy has historically been described as a bull’s eye maculopathy: a parafoveal ring of RPE degeneration sparing a foveal island typically occurring in symptomatic patients. However, using modern retinal imaging (particularly OCT), early parafoveal damage may be detected in asymptomatic individuals and characterized by outer retinal damage visible on OCT or by localized reduced sensitivity on automated visual field testing. Thus, the “textbook” description of hydroxychloroquine retinopathy, the bull’s eye maculopathy, should be remembered but not observed since earlier diagnosis using modern retinal imaging techniques should prevent its occurrence. However, bull’s eye changes may still be observed in patients who have developed retinal toxicity before the OCT era or who have not been screened. (Yusuf et al., 2022).

#### 3.2.1 Inner retina

There have been controversies about whether hydroxychloroquine damages the inner retina. Pasadhika et al. showed peripapillary retinal nerve fiber layer thinning in clinically evident retinopathy and selective thinning of the macular inner retina in eyes without clinically apparent fundus changes (Pasadhika and Fishman, 2010). However, another study on ganglion cell-inner plexiform layer (GC-IPL) thickness in long-term hydroxychloroquine users revealed no significant correlation between macular GC-IPL thickness and the cumulative dose of hydroxychloroquine (Lee et al., 2014). Topographic analysis of inner and the outer retinal thickness showed that the inner retina was not damaged in eyes with hydroxychloroquine retinopathy (de Sisternes et al., 2015). However, a recent report demonstrated inner retinal thinning in patients with advanced retinopathy (Kim et al., 2023). Further, there have been multiple cases with cystoid macular edema reported from patients with hydroxychloroquine retinopathy (Parikh et al., 2016; Hong et al., 2017; Kim et al., 2018). These cases were advanced retinopathy with RPE involvement in common; accordingly, a damaged retinal pigment epithelium and breakdown of the outer retinal barrier may have allowed accumulation of fluid within the retina, also involving the inner retina.

#### 3.2.2 Outer neural retina

**FIGURE 2:**
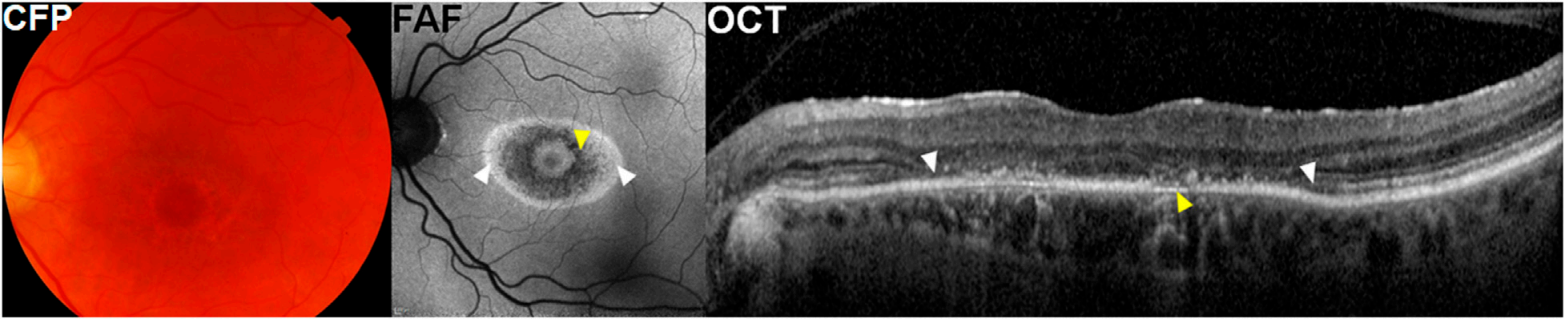
A representative case with parafoveal hydroxychloroquine retinopathy. Color fundus photographs (CFP) show parafoveal pigmentary changes. Fundus autofluorescence (FAF) images illustrate more prominent bull’s-eye macular lesion, represented as parafoveal rings of hypo autofluorescence (yellow arrowheads) and hyper autofluorescence (white arrowheads). Optical coherence tomography (OCT) images demonstrate photoreceptor loss (white arrowheads) and thinning of retinal pigment epithelium (RPE)/Bruch’s membrane complex (yellow arrowhead).

The characteristic findings of hydroxychloroquine retinopathy on OCT imaging include disruption or loss of the photoreceptor layers (Figure 2, white arrowheads) (Chen et al., 2010; Marmor, 2012; Marmor and Melles, 2014; Greenstein et al., 2015). Defects in the ellipsoid zone (EZ) are the most identifiable, since it is the most reflective and discernible layer amongst the photoreceptor layers on OCT imaging. As the disease advances, the EZ defect progresses and leads to the loss of all photoreceptor layers. Eyes with hydroxychloroquine retinopathy may be classified according to the topographical distribution of the most obvious photoreceptor damage, parafoveal, pericentral, or both. Eyes with parafoveal involvement show outer retinal damage in the inner parafoveal field (approximately 2°–6° eccentricity, around 500–1,500 μm from the foveal center), whereas those with pericentral involvement demonstrate outer retinal changes outside the parafoveal area, typically around the major vascular arcades.

Outer retinal thinning is another hallmark of hydroxychloroquine retinopathy on OCT imaging. This change usually occurs in the areas with photoreceptor defects (Chen et al., 2010). A recent study showed retinal thinning in a recognizable pattern (including parafoveal and pericentral) on OCT retinal thickness maps in eyes with early retinopathy (Kim et al., 2021). Inferior parafoveal or pericentral area may be of particular interest for early detection using thickness maps as previous studies showed that photoreceptor changes start inferiorly in the macula, spreading circumferentially to the superior area (Ahn et al., 2021; Kim et al., 2022). By generating total, inner, and outer retinal thickness maps, de Sisternes *et al.* demonstrated a characteristic parafoveal pattern of outer retinal thinning in eyes with parafoveal retinopathy (de Sisternes et al., 2015). Although it might be overlooked by a clinician’s qualitative evaluation of B-scan images, several authors reported cases of hydroxychloroquine retinopathy with abnormal visual fields showing no obvious photoreceptor or RPE defects but a subtle outer nuclear layer or photoreceptor layer thinning (Chen et al., 2010; Marmor, 2012; Marmor and Melles, 2014; Greenstein et al., 2015). Careful attention is required to evaluate the outer retinal thickness in cases of suspected retinopathy, as subtle thinning in the absence of obvious photoreceptor defects may be present in early disease.

#### 3.2.3 Retinal pigment epithelium

In eyes with hydroxychloroquine retinopathy, the RPE can show clinically relevant changes, as shown in Figure 2 (where attenuation and thinning of the RPE/Bruch’s membrane complex is shown [yellow arrowheads]). In severe cases with RPE defects, the choroid layer usually shows increased reflectance due to a reduction in optical attenuation by the RPE (choroidal hyper-transmission).

Fundus autofluorescence (FAF) is a noninvasive imaging modality that produces a density map of fluorophores in the RPE and can capture a wide area in a confocal plane within a single image (Yung et al., 2016). Hypo-autofluorescence on FAF may be due to reduced or absent lipofuscin, which may be caused by a loss of the RPE cells, such as in eyes with advanced hydroxychloroquine retinopathy. Typically, hypo-autofluorescence is observed in the area of outer retinal damage, either in parafoveal or pericentral areas, in eyes with severe disease.

Modern imaging data suggest that photoreceptors most likely degenerate first and RPE degeneration develops subsequently in hydroxychloroquine retinopathy. Photoreceptor changes precede RPE defects on OCT imaging and hypo-autofluorescence on FAF which signifies damage to or loss of the RPE (Marmor et al., 2016; Ahn et al., 2021).

In addition to the RPE, there have been a few reports on the choroid or choriocapillaris involvement in hydroxychloroquine toxicity. Ahn et al. showed choroidal and choriocapillaris thinning in eyes with hydroxychloroquine retinopathy (Ahn et al., 2017b). Studies on OCT angiography also showed evidences of choriocapillaris involvement, flow deficits or signal void zones in the choriocapillaris, in hydroxychloroquine retinopathy (Halouani et al., 2022).

### 3.3 Clinical classification of hydroxychloroquine retinopathy

The definition of hydroxychloroquine-induced retinal toxicity varies across studies (Table 3), in part because diverse diagnostic techniques - such as fundus photography, OCT, FAF, and multifocal electroretinography (mfERG)—are used to detect the disease. The earliest studies (prior to 2010) lacked an objective means of assessing retinal damage compared to the modern standard. For example, toxicity was typically defined as bull’s eye retinopathy on fundus photography or clinical examination, which we now understand as a severe and late manifestation of toxic retinopathy. Therefore, the definition of toxicity should be carefully considered before comparing the data from different epidemiologic studies on hydroxychloroquine retinopathy. Moreover, some differences in the subjective interpretation of objective tests may result in further variation. In the era of advanced retinal imaging, the classification of toxicity and disease severity relies on more objective, structural findings. Retinopathy is currently classified according to disease severity and/or pattern of retinopathy.

**TABLE 3:** Prevalence of hydroxychloroquine retinopathy studies using modern retinal imaging techniques. Note the wide variations in the definition of toxicity, diagnostic examinations and the risks characteristics of patients between studies, which impact on the estimates of disease prevalence amongst long-term users.

Year	Author	Study design	Country	Prevalence	Number of patients with HCQ retinopathy/included subjects	Duration of HCQ use (yrs, mean ± SD)	Definition of HCQ toxicity	Diagnostic examinations
2014	Melles et al	Retrospective	United States	7.5%*	177/2361	15.1 ± 5.5 (retinopathy)	Either characteristic VF changes or SD-OCT findings of retinal thinning and photoreceptor damage	Fundus photography, SD-OCT, HVF 10-2
2014	Browning et al	Retrospective	United States	10.4%	12/115	Not available	Based on the totality of the clinical evidence	SD-OCT, HVF 10-2, mfERG
2015	Jaumouillé et al	Prospective	France	6.5%	12/184	7.89 ± 7.21 (all)	2011 AAO guideline	SD-OCT, FAF, HVF 10-2, mfERG
2015	Lee et al	Retrospective	South Korea	4.1%	9/218	9.65 ± 2.57 (retinopathy)	Abnormal SD-OCT	SD-OCT, FAF, HVF 10-2 or 30-2
2015	Johnston et al	Retrospective	Canada	1.6%	2/126	9.8 (median)	Abnormalities in ≥2 of 4 tests	SD-OCT, fundus examination, HVF 10-2, visual acuity
2017	Eo et al	Retrospective	South Korea	2.9%	9/310	5.98 ± 3.37	Characteristic findings on OCT, abnormal autofluorescence on FAF, and decreased visual sensitivity or parafoveal scotoma on 10-2 visual field	SD-OCT, FAF, and automated perimetry
				5.2%*	9/174*			
2017	Ahn et al	Retrospective	South Korea	3.8%	24/630	13.7 ± 4.5 (retinopathy)	1) objective test abnormalities of the pericentral or parafoveal areas on FAF or mfERG in both eyes and 2) corresponding VF defects on HVF 10-2 or 30-2, based on 2016 AAO guideline	SD- and SS-OCT, FAF, HVF 10-2 or 30-2, mfERG
2020	Marshall et al	Prospective	United Kingdom	1.6%	14/869	15.9 ± 7.5	Two abnormal test results	OCT, FAF, HVF 24-2
				6.3%	55/869		One abnormal test result	
2020	Gobbett et al	Prospective	United Kingdom	0.6%	2/333	Not available	Two abnormal test results	Fundus photography, 10-2 HVF, OCT, FAF, and mfERG
2020	Dadhaniya et al	Cross-sectional	India	6.4%	7/110	13 ± 4.9	Ophthalmologist’s diagnosis based on visual field and SD-OCT abnormalities	Fundus examination, OCT, HVF 10-2 and 30-2

#### 3.3.1 Pattern of retinopathy (retinal phenotypes)

Published studies converge on the description of two distinct patterns of retinopathy: parafoveal and pericentral. These descriptions indicate the predominant location of the retinal toxicity. However, not all cases fit this apparent binary classification. One pattern may not be clearly distinct from the other and advanced retinal toxicity may have both parafoveal and pericentral involvement, regardless of the initial disease distribution.

**FIGURE 3:**
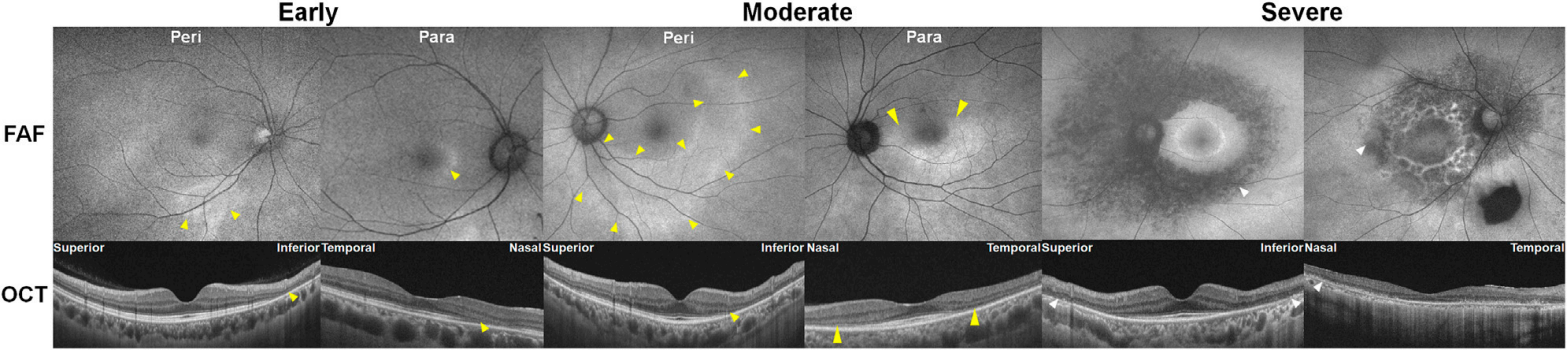
Classification of hydroxychloroquine retinopathy according to the pattern and severity of retinopathy. Eyes with hydroxychloroquine retinopathy can be divided into those with early (patchy photoreceptor defects without retinal pigment epithelium [RPE] involvement), moderate (photoreceptor damage constituting a partial or full ring [>180°] without RPE involvement), and severe (combined RPE damage) retinopathy. Yellow and white arrowheads indicate photoreceptor (represented as photoreceptor loss on optical coherence tomography [OCT] and hyper autofluorescence on fundus autofluorescence [FAF]) and retinal pigment epithelium damages (shown as an attenuated and thinned RPE/Bruch’s membrane complex line on OCT and hypo autofluorescence on FAF), respectively. Para = parafoveal retinopathy; Peri = pericentral retinopathy.

As shown in Figure 3, parafoveal retinopathy is defined as outer retinal defects 2°–6° from the fovea. Classically, bull’s eye maculopathy describes a ring of parafoveal RPE degeneration that spares a foveal island. Some patients demonstrate initial damage in a more peripheral distribution near the major retinal vascular arcades. This predominantly extramacular retinopathy is classified as pericentral retinopathy, as shown in Figure 3. In eyes with both patterns, parafoveal and pericentral, a mixed pattern of retinopathy is said to exist. Accordingly, in most studies on hydroxychloroquine retinopathy, the eyes were grouped into two or three (parafoveal, pericentral, and mixed) groups, based on the topographical distribution of retinal damage.

Interestingly, ethnic groups vary in the predominant distribution or pattern of retinopathy. In a large case series of 201 patients from diverse ethnic backgrounds, pericentral retinopathy was observed in 50% of Asian patients but only in 2% of Caucasian patients (Melles and Marmor, 2015). Another study showed that almost 90% of Korean patients with hydroxychloroquine retinopathy had a pericentral pattern (Lee et al., 2015). In contrast, other populations demonstrate a predominantly parafoveal pattern. This pattern was more prevalent in Caucasian populations (parafoveal: pericentral = 101:2) than in black (11:3) or Hispanic patients (17:1), although the difference was not significant in the relatively small studies which described them (Melles and Marmor, 2015).

Disease severity at the time of detection varies according to the pattern of retinopathy (Melles and Marmor, 2015). Retinopathy was typically detected at an earlier stage in eyes with parafoveal disease (32.0% early and 27.5% severe) when compared to pericentral disease (4.2% early and 62.5% severe). It is unclear as to whether this represents a bias in diagnostic testing which favours the detection of macular disease (i.e., standard field OCT imaging, visual field testing). As pericentral retinopathy is common among Asians, this population is more likely to be diagnosed with severe disease (Melles and Marmor, 2015). However, it should be noted that “Asian” in the initial report by Melles and Marmor describes a predominantly East Asian population, rather than Central or South Asian. Additional data are required in the other Asian ethnic groups to conclude the association between the Asian population more generally, and pericentral retinopathy.

The classification of disease according to the pattern of retinopathy has several clinical implications. Screening tests, such as OCT and automated perimetry, vary in the field of coverage. For example, retinopathy may not be detected by standard 6-mm-long OCT horizontal and vertical line scans in some eyes with the pericentral disease (Ahn et al., 2017a). Therefore, clinicians should select an appropriate protocol depending on the common pattern of retinopathy prevalent in the population being screened. However, given that pericentral disease is also present in non-Asian patients, screening protocols should ideally include at least one test that can detect pericentral disease in any population (Yusuf et al., 2021b).

Several studies have recommended wide-field imaging and wider visual field tests to detect pericentral retinopathy (Lee et al., 2015; Melles and Marmor, 2015; Ahn et al., 2017a; Ahn et al., 2020). The 2016 guideline of the American Academy of Ophthalmology (AAO) also recommended wider-angle scans or wider test patterns for Asian patients (Marmor et al., 2016). Furthermore, the Royal College of Ophthalmologists guideline recommends widefield FAF imaging for all patients to capture pericentral disease (Yusuf et al., 2021b). Although the necessary field of imaging for the early detection of pericentral retinopathy has not been fully determined, retinal imaging of 50° or greater would capture the retinopathy in most cases as it covers the areas around the major vascular arcades. Humphrey visual field (HVF) or FAF protocols specifically adapted to Asian patients have not yet been standardized, although the AAO guideline suggested 24-2 or 30-2 for HVF (Marmor et al., 2016). The Royal College of Ophthalmologists (RCOphth) guideline (2020) recommends an HVF test strategy appropriate to the anatomical location of the objective structural damage (Yusuf et al., 2021b). Using OCT, Ahn et al. suggested 12 × 9-mm volume scans or wide-field (12-mm) radial scans as these protocols resulted in 100% sensitivity for the detection of retinopathy in Asian patients (Ahn et al., 2017a).

#### 3.3.2 Disease severity

Eyes with hydroxychloroquine-induced retinal damage can be classified as early, moderate, or severe based on the extent of outer retinal damage (Marmor, 2012). Representative cases for these severity grades are presented in Figure 3. Early and moderate retinopathy are defined as patchy photoreceptor defects (≤180° around the fovea) and ring (>180°) of photoreceptor defects, respectively. Eyes with combined RPE defects (observed as hypo-autofluorescence on FAF) are designated as severe retinopathy.

This classification of hydroxychloroquine retinopathy has been used in multiple studies, as the severity of retinopathy can indicate prognosis after drug cessation. For example, Pham et al. showed stable retinopathy, in terms of outer retinal structure and visual function, for up to 9 years after hydroxychloroquine cessation in eyes with early and moderate retinopathy but progressive retinopathy for up to 20 years after drug cessation in cases of severe retinopathy (Pham and Marmor, 2019). Other studies also showed an association between retinopathy progression and severity (Mititelu et al., 2013; Ahn et al., 2019a; Ahn et al., 2019b). This suggests that the severity of hydroxychloroquine retinopathy should be determined at the time of diagnosis for its predictive value on the risk of progression and for clinical decision-making to discontinue drug administration.

However, Allahdina et al. suggested a new staging system based on OCT findings: Stage 1, subtle changes confined to the parafoveal region; 2, definite localized changes in the parafovea; 3, extensive parafoveal changes; and 4, foveal involvement. Although this system can be applied mainly for the classification of eyes with parafoveal retinopathy, these stages were functionally correlated with visual acuity, visual field test results, or mfERG and significantly associated with the degree of further progression (Allahdina et al., 2019).

**FIGURE 4:**
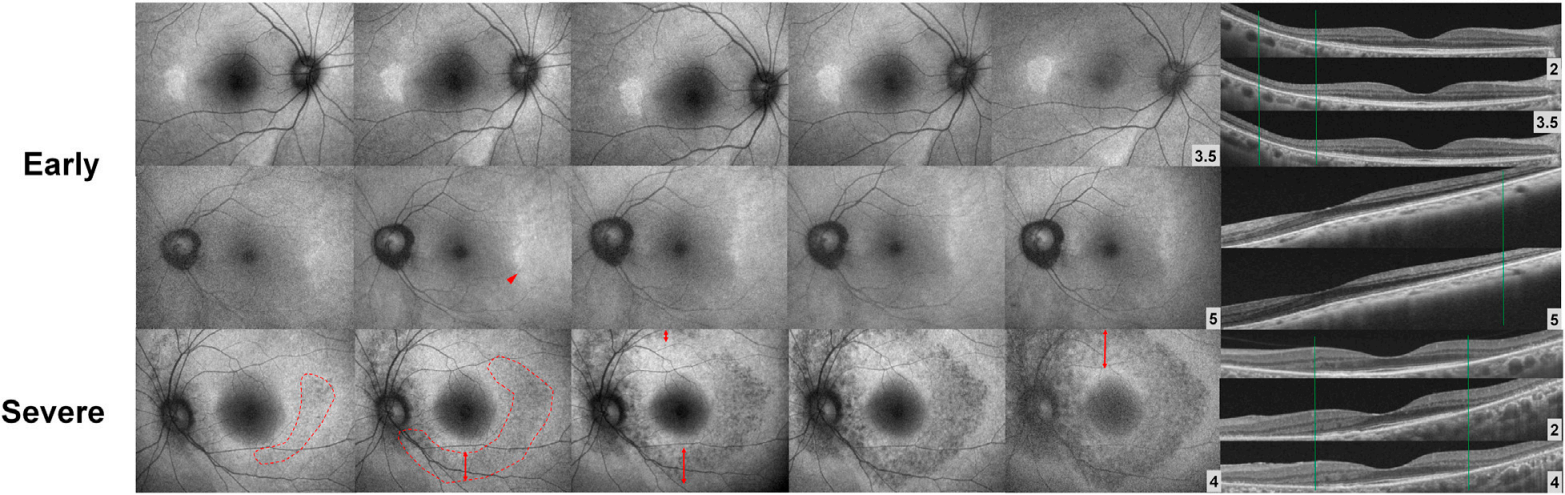
Fundus autofluorescence (FAF; left) and optical coherence tomography (OCT; right) images over time after drug cessation in selected early and severe eyes with hydroxychloroquine retinopathy. The lesions in FAF and OCT images are stable in early (top) cases, whereas the severe case (bottom) shows continuous progression threatening to the macula. (Modified from Ahn et al. Ophthalmology. 2021; 128:889-898).

Although the risk of severe visual impairment in early, moderate, and severe retinopathy has not been reported in a large number of patients, the risk of severe visual impairment in eyes with parafoveal severe retinopathy is intuitively higher since photoreceptor loss can progress to involve the foveal center. Irreversibility of retinal damage is also associated with the severity of retinopathy; for example, eyes with severe retinopathy show a continuous progression of retinopathy, whereas those with less severe forms of retinopathy show stable outer retinal defects or even photoreceptor recovery (Figure 4) (Mititelu et al., 2013; Pham and Marmor, 2019; Ahn et al., 2021). Based on previous reports, the transition to severe retinopathy (with RPE loss) appears to form a threshold beyond which progressive damage continues after drug cessation. As there is currently no treatment that can restore the loss of visual function caused by the drug, early recognition (before the severe stage) and management is crucial, as this minimizes the risk of blindness by reducing the risk of disease progression. Further, the severity-dependent manner of disease progression highlights the significance of clinical classification of disease severity in hydroxychloroquine retinopathy.

## 4 Epidemiology

### 4.1 Prevalence estimates

The estimated prevalence of toxic retinopathy in users of hydroxychloroquine and chloroquine reported in the literature varies greatly, principally owing to differences in the definition of toxicity, the sensitivity of the tests used to identify toxicity, and the risk characteristics of the screened population (i.e., daily dose and duration of exposure). Furthermore, variability in the subjective interpretation of test results between clinicians is likely. It should be noted that studies in this field use the term “prevalence” to mean the frequency of retinopathy within at-risk groups (hydroxychloroquine users, or long-term hydroxychloroquine users) rather than as a population-based term.

Table 3 compares the prevalence of hydroxychloroquine retinopathy in studies undertaken in the OCT era. Studies prior to the OCT era found a prevalence of retinopathy of less than 0.5% using different sets of diagnostic tests for detection (Morsman et al., 1990; Levy et al., 1997; Mavrikakis et al., 2003). Historically, there were only 18 cases of hydroxychloroquine-induced retinal toxicity across 11 published reports from 1963 to 1989 (Mikkelsen, 1979; Tobin et al., 1982; Mackenzie, 1983a; Rynes, 1983; Finbloom et al., 1985).

In 2011, the AAO revised its guidelines, suggesting an increased risk of toxicity with a longer duration of therapy and highlighting the improved screening tools (Marmor et al., 2011). The recommendations advocated the use of OCT and FAF, where available, to provide objective evidence of toxicity (Marmor et al., 2011), which were not widely available before. In 2014, Melles and Marmor evaluated the risk of retinopathy in 2,361 patients who had used hydroxychloroquine for more than 5 years, using the definition of toxicity of *either* characteristic visual field changes *or* spectral-domain optical coherence imaging findings of retinal thinning and photoreceptor damage (i.e., only one abnormal test result was required) (Melles and Marmor, 2014). This study identified a prevalence of toxicity of 7.5% in this group overall, increasing to 20%–50% after 20 years of exposure (Marmor and Melles, 2014). These estimates of toxicity were far higher than those previously considered, partly because of the more sensitive detection by OCT, relatively long mean duration of hydroxychloroquine use within the study participants (mean duration of therapy was 15 years), and more frequent prescription of high doses before a new safe dose was defined by the same study (Melles and Marmor, 2014).

In a French cohort screened prospectively according to the AAO guidelines from 2011, 12 cases of toxicity were identified from 184 screened individuals, with a prevalence of 6.5% (Jaumouille et al., 2015). In this study, visual field testing and spectral domain-OCT (SD-OCT) imaging were considered more sensitive than FAF imaging in detecting the early stages of disease (Jaumouille et al., 2015), a finding also reported by Cukras et al. (Cukras et al., 2015). A large, prospective audit of 1,967 screening attendances from a single-center in the UK identified a prevalence of retinopathy of 6.3% based on at least one abnormal test result, and 1.6% based on the RCOphth criteria of two abnormal tests for definite toxicity (Marshall et al., 2021). The mean duration of therapy was 15.9 years in the group with more than 5 years of hydroxychloroquine exposure in this study. The prevalence in the group with more than 5 years of hydroxychloroquine exposure (6.3%) was comparable with that reported by Melles and Marmor (7.5%). Two further large, published audits within the UK identified a similar frequency of retinopathy using an identical definition of toxicity (aggregated frequency of 1.47% across the three studies (Gobbett et al., 2021; Alieldin et al., 2022)).

### 4.2 Risk factors and management

#### 4.2.1 Daily dosing

The most critical risk factors for retinal toxicity are the daily dose and duration of exposure. Following the discovery of the relative safety of hydroxychloroquine over chloroquine, investigators focused on identifying a safe daily dose of hydroxychloroquine. However, there is probably no absolutely safe dose, as retinal damage occurs due to the combined effects of dose and duration of hydroxychloroquine use (Melles and Marmor, 2014).

Some authors have suggested that daily dose is more predictive of retinal toxicity than cumulative drug exposure, presumably because it was considered that daily dose more accurately predicted drug accumulation within susceptible tissues (Mackenzie, 1983a;b). In qualifying the understanding of daily dose, some authors recommended that daily dosing be based on ideal (or lean) body weight rather than actual body weight (Mackenzie, 1983a; Easterbrook, 1999). The concern was that patients may be receiving an excessive dose based on actual body weight since the drug may be sequestered in fat. However, there is no clear evidence of systemically administered hydroxychloroquine accumulation in fatty tissues. Animal studies with extremely high doses showed that the concentrations of hydroxychloroquine were similar in the skin, fat, and muscle (McChesney et al., 1967; McChesney, 1983). The calculation of ideal body weight is more complex than using actual body weight since there are several formulae used for calculation, and the parameter requires the measurement of height (Melles and Marmor, 2016).

As the daily dose limits relate not to efficacy, but the risk of retinal toxicity, safe daily dosing should be defined in relation to the risk of retinal toxicity. In 2014, Melles and Marmor found that the risk of toxicity varied with daily dose in patients taking a dose of >5 mg/kg/day of absolute body weight at greatest risk (odds ratio: 5.67). The prevalence of toxicity in patients taking 4–5 mg/kg/day of hydroxychloroquine for ≤10 years was below 2%, although toxicity was still detected in some patients taking 3 mg/kg/day for long periods or with other risk factors. No daily dose was completely without risk with risk estimates also accounting for the duration of use (Melles and Marmor, 2014). When the relationship between weight and dosage was carefully analyzed, ROC curves showed that real weight was a better predictor of risk and gave almost equal predictions for individuals with large intervals in body mass index (BMI) from 15 to 35 kg/m^2^. This led to re-examination and rejection of the early hypothesis that hydroxychloroquine was selectively absorbed in fat and supported the recommendation that real rather than ideal weight should be used to calculate the daily dose. At doses of less than 5 mg/kg/day of absolute body weight, the risk of retinopathy was approximately half of that in the greater than 5 mg/kg/day daily dosing group (Melles and Marmor, 2014). This case-control study was the largest and most influential, which examined the risk factors for hydroxychloroquine retinopathy, and daily dosing based on actual body weight that is currently more widely accepted as the standard (Marmor et al., 2016; Fanouriakis et al., 2019). Furthermore, the use of actual body weight simplifies the implementation of safe dosing in the clinic. Accordingly, the revised 2016 AAO recommendations advise a safe dosing limit based on actual body weight (<5 mg/kg/day), which replaced the 2011 recommendations that were based on ideal body weight (<6.5 mg/kg/day). These safe dosing limits were also incorporated into the RCOphth recommendations (2018 and 2020) (Yusuf et al., 2018; Yusuf et al., 2021b). Some suggest a strategy to prescribe a lower dose of actual and ideal body weight calculations or use ideal weight for obese patients (Browning and Lee, 2018); however, no convincing evidence has been published to suggest that obese individuals are at higher risk of hydroxychloroquine retinopathy using conventional dosing than normal individuals.

**FIGURE 5:**
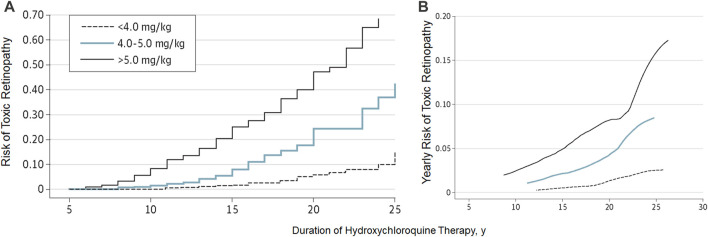
Cumulative risk **(A)** and yearly risk **(B)** of hydroxychloroquine retinopathy. This Kaplan-Meier curve shows different cumulative risk over time (and also yearly risk) according to the daily dose. (Reprinted with permission from Melles RB, Marmor MF. JAMA Ophthalmol 2014; 132:1453-60).

Note that the risk estimates above and the Kaplan-Meier curve (Figure 5) on the cumulative risk of retinal toxicity represent a population risk with the indicated dosage and duration of use. However, these data cannot predict the risk for a given individual. This is an important consideration when advising patients on the risk of continued hydroxychloroquine therapy, particularly after a long duration of use. Figure 5B shows the smoothed hazard estimates of the risk of developing retinopathy in the ensuing year if the examination is normal. Even after 20 years, the risk of developing retinopathy in 1 year is less than 4% (compared to a population risk of approximately 20% after 20 years if never previously examined), and this justifies the continued use of daily doses less than 5 mg/kg/day under adequate screening care.

Finally, it is critical to understand that the dose alone (as discussed above) is not sufficient to determine risk or safety. The effects of different dose levels are affected by the duration of use, and whilst a high daily dose or long duration are critical risk factors, the actual risk is a function of the two risk factors (Figure 5). Keeping the daily dose under <5 mg/kg confers a low risk for up to 10 years; however, beyond this dose, the risk increases. Higher daily doses further increase the risk, particularly between 5 and 10 years of treatment. A very low dosage, if found to be clinically efficacious, would lower the risk and extend the duration of safe usage further.

#### 4.2.2 Duration

Historically, the duration of exposure to hydroxychloroquine has been thought to be a risk factor for developing retinopathy. A study of 3,995 users of hydroxychloroquine who self-reported adverse effects identified a five-fold increased risk of toxicity in patients who had more than 7 years of therapy than those who had a shorter course of treatment (Wolfe and Marmor, 2010). Melles and Marmor showed in a case-control study of 2,361 individuals taking hydroxychloroquine comprising 177 patients with toxicity. The risk of retinopathy increased with the duration of use (odds ratio of 3.22, with a duration of over 10 years) (Melles and Marmor, 2014). Furthermore, a detailed analysis of the effect of duration of therapy in groups with different daily doses identified that increasing duration of therapy predicted an increased risk. There was an insignificant risk in the first 5 years of therapy, although, in the highest dose group (>5 mg/kg/day), the risks at 10, 15, and 20 years were approximately 10%, 20%, and 40%, respectively (Melles and Marmor, 2014). These are the most comprehensive data available to define the risk of toxicity with the duration of hydroxychloroquine therapy.

Interestingly, clinical trials that evaluated the adjuvant use of high-dose hydroxychloroquine as an inhibitor of autophagy have identified toxic retinopathy occurring after a much shorter treatment duration than that with conventionally used doses (Poklepovic and Gewirtz, 2014). A study evaluating the use of hydroxychloroquine (1 g daily) with erlotinib for non-small cell lung cancer (Goldberg et al., 2012) found toxic retinopathy in two of seven patients at 11 and 17 months of therapy (Leung et al., 2015). This finding confirms that both daily dose and duration combine to influence the risk of toxicity.

The total cumulative dose of antimalarials is often evaluated as a candidate risk factor for the risk of toxic retinopathy. However, it is often more challenging to calculate the total cumulative dose than the daily dose. The cumulative dose/kg of weight can produce a single combined value to predict risk; however, some information is lost. For example, it has become apparent that a patient taking a lower dose for a longer duration may be exposed to a different risk compared to a patient taking a higher dose for a shorter duration, even if the total cumulative dose is equal. This difference is related to the drug pharmacokinetics, which in part due to its long half-life, reaches a steady state after approximately 6 months (Tett et al., 1989). However, the cumulative dose of hydroxychloroquine is associated with mfERG changes in two separate studies (Lai et al., 2006; Lyons and Severns, 2007). Furthermore, for patients with a cumulative dose of greater than 20 gm/kg, the odds ratio of toxicity was 8.13, the strongest of any risk factor identified in the study (Melles and Marmor, 2014).

As noted at the end of Section 4.2.1, the duration as a risk factor must always be considered in the context of daily dose. The Kaplan-Meier curve (Figure 5) must be used to accurately estimate the effects of duration on the risk of toxicity under different dosage regimens.

#### 4.2.3 Other risk factors

##### 4.2.3.1 Renal impairment

Renal impairment has been considered a possible risk factor for toxicity (Mackenzie, 1983a; Bernstein, 1991), and is considered in early screening recommendations (Levy et al., 1997; Mavrikakis et al., 2003). Melles and Marmor identified that stage 3 kidney disease or worse (defined by a glomerular filtration rate of less than 60 mL/min/1.73 m^2^) conferred an increased risk of toxic retinopathy (odds ratio: 2.08) (Melles and Marmor, 2014). Furthermore, the risk of retinopathy appeared to increase as renal function deteriorated (Melles and Marmor, 2014). Therefore, the recent AAO and RCOphth screening recommendations support renal impairment as an additional risk factor and suggest the institution of annual screening in hydroxychloroquine users with stage 3 renal failure (Marmor et al., 2016; Yusuf et al., 2018; Yusuf et al., 2021b). Adjustment of daily dose might reduce risk in hydroxychloroquine users, but this has not yet been validated. The potential role of serum hydroxychloroquine levels in refining daily doses in patients with renal failure requires further investigation.

##### 4.2.3.2 Tamoxifen

Drugs that confer additional toxic effects on the retina or interact with hydroxychloroquine may shorten the interval to toxicity. For example, a patient taking conventional doses of hydroxychloroquine has been reported to have retinal toxicity after concomitant treatment with docetaxel and cyclophosphamide for breast cancer (Sharma et al., 2019). This suggests that the observation of accelerated toxicity may be partly due to a drug interaction with one or more chemotherapeutic agents, rather than simply using the high doses of hydroxychloroquine in studies of autophagy inhibition in oncology.

Tamoxifen is often administered to patients with estrogen receptor-positive breast cancer for 5–10 years after diagnosis. Tamoxifen retinopathy was first described in 1978 in four patients receiving high-dose therapy for a duration greater than 1 year (Kaiser-Kupfer and Lippman, 1978; Kaiser-Kupfer et al., 1981). The retinal phenotype was of crystalline retinopathy with refractile deposits in the paramacular area, which were histologically localized to the inner retina (nerve fiber and inner plexiform layers) (Kaiser-Kupfer et al., 1981). Numerous reports of tamoxifen retinopathy were published over the following 20 years, although the prevalence of toxicity among low-dose users was considered low, partly as OCT imaging was unavailable (Gorin et al., 1998). Kim et al. identified that 30 out of 251 tamoxifen users (12%), with greater than 2 years of exposure, had evidence of fundus and OCT changes consistent with tamoxifen retinopathy. Twelve patients had foveal cavitations, four had refractile crystalline deposits, and 16 had both (Kim et al., 2020). All patients were taking 20 mg of tamoxifen per day and only 8 of the 30 individuals were symptomatic. Furthermore, multiple regression analyses identified that hyperlipidemia and increased BMI were associated with a greater risk of retinopathy among users (Kim et al., 2020).

Although both tamoxifen and hydroxychloroquine had been independently associated with retinal toxicity, tamoxifen was found to increase the risk of toxicity in hydroxychloroquine users (Melles and Marmor, 2014). The odds ratio of retinal toxicity in patients taking tamoxifen was 4.59 (95% confidence interval 2.05–10.27) (Melles and Marmor, 2014). However, no further studies have yet confirmed the increased risk of hydroxychloroquine retinopathy in tamoxifen users. Nevertheless, this finding was incorporated into the recommendations for retinopathy screening in the US and United Kingdom (Marmor et al., 2016; Yusuf et al., 2018; Yusuf et al., 2021b). It is assumed that there is a synergistic toxic effect on the retina when the two drugs are taken concurrently, although the molecular basis of such synergy is unclear.

##### 4.2.3.3 Concomitant ocular disease

Concomitant ocular disease in hydroxychloroquine users is of interest and relevance for several reasons:

Any concomitant ocular disease (including glaucoma and media opacities) may limit the ability of screening procedures to detect changes or apportion any change to the toxic effects of hydroxychloroquine.

Patients with a pre-existing retinal disease may have a less structural and functional reserve and an additional toxic insult may not be tolerated, although this remains unproven.

Patients with SLE and RA, the major treatment indications for hydroxychloroquine use, are typically diagnosed before the age of 40 years for SLE and between 30 and 60 years for RA. Therefore, the onset of disease and hydroxychloroquine initiation is before the age at which macular and retinal disorders become more prevalent in the population (e.g., age-related macular degeneration, diabetic retinopathy, retinal vein occlusions, epiretinal membrane). Any retinal co-pathology could exist in new initiators of hydroxychloroquine, although unlikely in any given individual and less likely in young patients. Therefore, a baseline screening visit aims to establish whether the macula is structurally normal (or likely to interfere with screening procedures), and if not, whether there is a functional deficit in visual field testing which may be useful for later comparison. The use of hydroxychloroquine in patients with known macular pathologies may be considered on a case-by-case basis. The ophthalmologist should inform the prescribing physician if reliable screening for retinopathy may not be possible. If screening cannot be undertaken, an alternative therapy might be sought unless the patient agrees prospectively to accept that risk. There are no data in the literature regarding the detection of disease progression in hydroxychloroquine retinopathy in the presence of ocular co-pathologies. Furthermore, the disease for which hydroxychloroquine is indicated might have an independent effect on the results of a screening test. For example, SLE has been associated with choroidopathy and drusen-like deposits, independent of hydroxychloroquine use (Gharbiya et al., 2006; Invernizzi et al., 2017; Braga et al., 2019). Long-term treatment of RA may reduce the incidence of age-related macular degeneration (McGeer and Sibley, 2005).

There is currently no evidence to support the hypothesis that patients with the pre-existing macular disease may be more susceptible to the toxic effects of hydroxychloroquine. Further, there would be different risks for patients with inner pathology compared to those with outer retinal pathology as the primary site of retinal damage caused by hydroxychloroquine is the outer retina. For example, the patients with epiretinal membrane, a common macular pathology, might show similar risk to those without any macular pathology in terms of retinopathy development. Most importantly, hydroxychloroquine is a valuable, inexpensive, and effective drug for treatment of several rheumatologic and inflammatory diseases; accordingly, the presence of preexisting macular disease does not require switching from hydroxychloroquine to alternative drugs. However, the toxic effects of hydroxychloroquine should be carefully assessed and discriminated from progression of pre-existing maculopathy.

##### 4.2.3.4 Idiosyncratic and genetic factors

Despite these population risk estimates, it is observed that some individuals exhibit hydroxychloroquine retinopathy at 6 years at the recommended daily doses, while others remain disease-free after 35 years. Some unknown factors or modifiers affect susceptibility to hydroxychloroquine toxicity. These modifiers may be environmental, related to individual differences in pharmacokinetics and drug metabolism, or occur as a result of the effects of unidentified genetic variants. A small study showed that two out of 8 patients with chloroquine/hydroxychloroquine retinopathy had heterozygous *ABCA4* missense mutations previously associated with Stargardt disease (Shroyer et al., 2001). However, carrier frequencies of ABCA4 variants are high and may alone explain these findings. Another report also showed one case with heterozygous variant in USH2A (Katsman et al., 2017), which again may be due to expected sequence variations in a large gene. However, later and larger genetic analyses did not confirm the association (Grassmann et al., 2015; Mack et al., 2020). This remains a largely unknown territory, and larger studies are required to identify risk alleles for hydroxychloroquine retinopathy.

## 5 Diagnostic techniques and screening

Diagnostic tests for hydroxychloroquine retinopathy can be classified as structural or functional. As the most recent guidelines from the AAO recommend “at least one objective test confirming subjective findings” for the diagnosis of hydroxychloroquine retinopathy, the screening tests need to be further discriminated based on the degree of subjectivity. It should be noted that “objective tests” by these descriptions involve subjective assessment by clinicians. Since the gold standard used for evaluating sensitivities/specificities varies enormously among different studies and investigators with different degrees of experience, numerical comparisons (sensitivity, specificity) among tests might be of limited value. However, OCT, in particular, provides the most specific information as certain OCT features in a long-term hydroxychloroquine user may be considered almost pathognomonic for hydroxychloroquine toxicity. In contrast, amsler grid, color vision testing, and color fundus photography are no longer recommended as standard objective screening tests for hydroxychloroquine retinopathy. This is mainly due to the lack of sensitivity of these tests in the detection of early retinopathy.

### 5.1 Functional measures

#### 5.1.1 Visual fields

An automated visual field test may be used to document functional abnormalities caused by hydroxychloroquine toxicity. The revised recommendations of AAO, published in 2016, designated the automated visual field as the only subjective test among the four recommended tests (OCT, FAF, visual field testing and mfERG; Table 4) for hydroxychloroquine retinopathy. Therefore, visual field testing is one of the key standard tests for the diagnosis of hydroxychloroquine retinopathy. However, as this is a “subjective” test, the results can be variable and unreliable due to patient compliance (Marshall et al., 2021), with a known learning effect on repeated evaluation, often requiring repetition to ensure that subtle abnormalities are repeatable. Furthermore, abnormal findings should be correlated topographically with objective findings obtained by SD-OCT, FAF, and mfERG for confirmation before a diagnosis of hydroxychloroquine retinopathy is made.

**TABLE 4:** Observed findings from currently recommended tests used for screening of hydroxychloroquine retinopathy.

Examinations	Characteristics	Findings	References
Optical coherence tomography (spectral-domain or swept-source)	Objective, structural	Disruption or loss of the photoreceptor lines with or without RPE thinning or attenuation in the parafoveal (±central) or pericentral area	Browning (2014), Marmor et al. (2016), Ahn et al. (2021)
Fundus autofluorescence	Objective, structural	Hyper- or hypo-autofluorescence in the parafoveal (±central) or pericentral area	Marmor et al. (2016), Ahn et al. (2020)
Multifocal electroretinogram	Objective, functional	Amplitude reduction and prolonged implicit time in areas with retinal damage (parafoveal or pericentral ring response reduction or generalized reduction in advanced eyes)	Tsang et al. (2015), Marmor et al. (2016)
Standard automated perimetry	Subjective, functional	Central/parafoveal or pericentral scotomata with patchy (early), ring (typical), or constricted (advanced) pattern in Humphrey 10-2 or wider tests	Anderson et al. (2011), Browning (2014), Marmor et al. (2016), Ahn et al. (2021)

**FIGURE 6:**
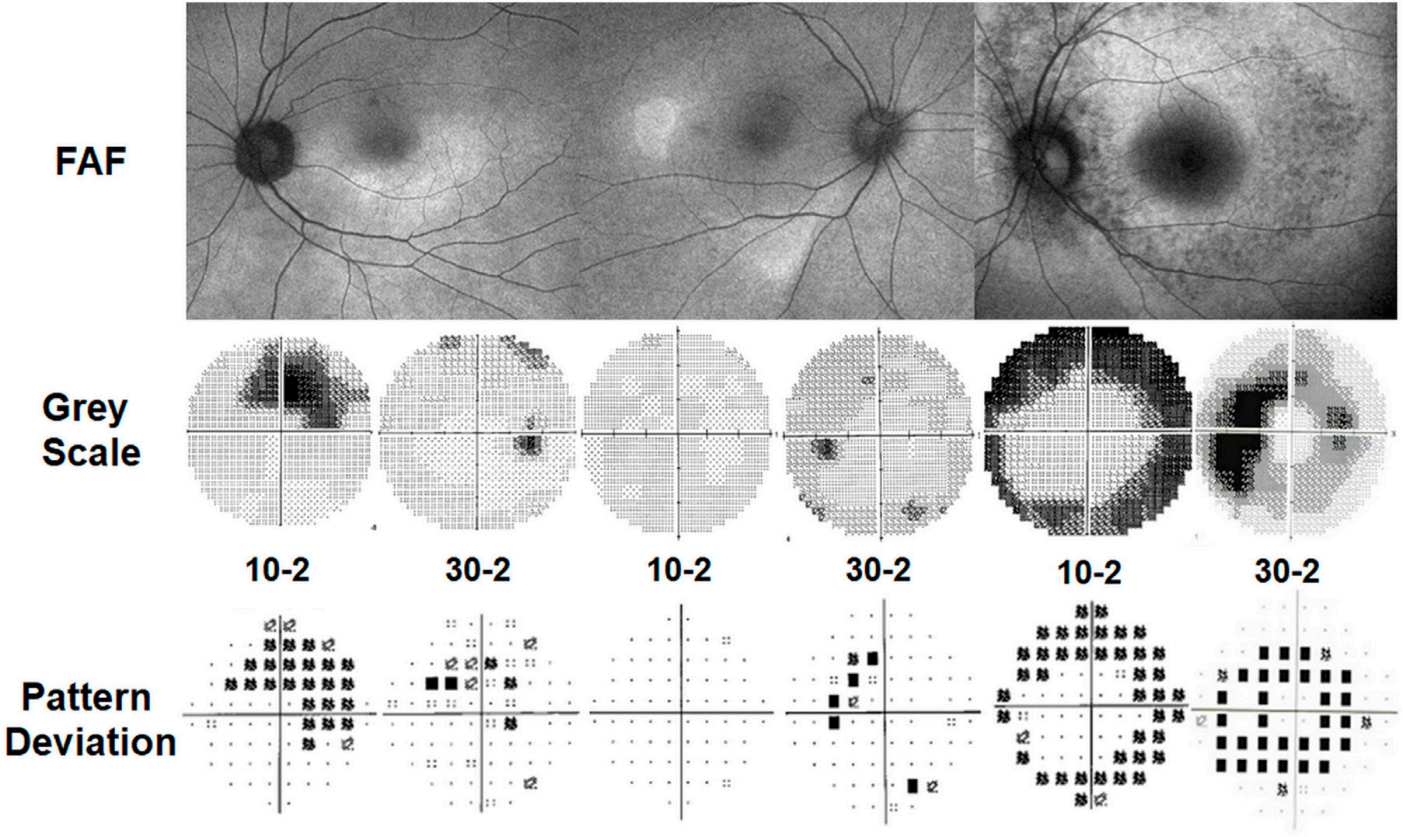
Automated visual field test results in patients with parafoveal (left) and pericentral (middle and right) hydroxychloroquine retinopathy. Numbers (10-2 or 30-2) indicate protocols used for the test. (Left) A case with moderate parafoveal retinopathy. The 10-2 field shows largely defined (partial ring) scotoma, while 30-2 shows less remarkable finding. (Middle) A case with early pericentral retinopathy. The 10-2 field shows normal field; 30-2 shows some patchy losses beyond 20°. (Right) A case with severe pericentral retinopathy. 10-2 shows constriction now extending in to about 5°; 30-2 shows ring-shaped losses sparing the central 10°.

The most recognizable pattern of visual field abnormality in patients with moderate to severe hydroxychloroquine retinopathy (Table 4) is a complete or incomplete ring scotoma, as shown in Figure 6. In eyes with early retinopathy, photoreceptor defects in the inferior and temporal retina correspond to a superonasal visual field defect on automated visual field testing (Figure 6, middle). Findings in early disease may appear non-specific and often require repeated visual field testing for confirmation. In advanced retinopathy (Figure 6, right), constricted visual fields with central sparing may be observed (Anderson et al., 2011).

Investigators have evaluated different visual field protocols and stimulus patterns in the evaluation of hydroxychloroquine retinopathy. Some clinicians favor visual field testing using red-colored stimuli, as they appear more sensitive. However, there is considerably more noise and the rates of false positives are higher, as the human eye is less sensitive to red than white (Anderson et al., 2011; Marmor et al., 2013). Furthermore, pattern deviation plots are not available with red fields. Most clinicians prefer white fields that are more stable and are widely recommended and available (Marmor et al., 2016). The 10-2 visual field protocol has been most commonly used for the screening of hydroxychloroquine retinopathy given that retinopathy is classically centered on the parafovea (2°–6° from the fovea). However, in pericentral retinopathy, which is common in some Asian populations, the 10-2 test usually does not capture focal pericentral defects that occur in eyes with early retinopathy (Figure 6, middle). Accordingly, this protocol may lead to missed or late diagnosis in patients with pericentral retinopathy. The 24-2 or 30-2 tests are more desirable to identify visual field defects associated with focal pericentral photoreceptor defects; however, it is still necessary to perform 10-2 field testing and often to repeat fields as patients vary in the pattern of their retinopathy. The most useful protocol for visual field testing in patients at risk of retinopathy in different populations has not yet been determined, and it is hoped that manufacturers of visual field analyzers will devise a rapid combined central and pericentral field test with sufficient sensitivity for use in all patients.

The interpretation of VF results for hydroxychloroquine retinopathy screening also requires consideration. Reliability indices should be scrutinized before any attempt to interpret the visual field plot; unreliable or inconsistent responses require repeated testing. The pattern deviation plot provides consistent results on visual field defects compared with the grayscale map (Anderson et al., 2011). Therefore, objective data are required to confirm visual field abnormalities. It has been reported that up to 10% of patients with hydroxychloroquine retinopathy may have characteristic visual field deficits without clear abnormalities on OCT imaging (Marmor and Melles, 2014). However, even in such cases, careful examination of the outer nuclear layer thickness in the corresponding regions should be undertaken, as subtle thinning may be present without clear qualitative outer retinal abnormalities. Moreover, other studies have confirmed cases of retinopathy with characteristic OCT changes and mfERG abnormalities with normal visual fields (Garrity et al., 2019).

A large UK audit of hydroxychloroquine screening outcomes, conducted according to standardized RCOphth monitoring recommendations (2018), identified that visual fields were unreliable in 33% of cases, among which 24.9% were of poor quality (Marshall et al., 2021). A further audit of 333 individuals identified that 17% of visual field tests were unreliable (Gobbett et al., 2021). The RCOphth amended their recommendations in 2020 by reorganizing the recommended tests such that all individuals receive SD-OCT and FAF imaging (Yusuf et al., 2021b). In this revised guideline, visual fields are reserved for those individuals who demonstrate an objective structural abnormality consistent with hydroxychloroquine retinopathy; the visual field protocol can then be matched to the location of the structural abnormality (30-2 for a pericentral defect). However, automated visual field tests, widely available in ophthalmology clinics, are currently used as the key screening tools for retinopathy worldwide and cannot be replaced as subjective, functional tests.

#### 5.1.2 Microperimetry

Microperimetry is another functional test that integrates standard automated perimetry with real-time fundus imaging (Pfau et al., 2020). By providing precise localization of functional defects, this modality has the potential to match the defects topographically with structural defects in eyes with hydroxychloroquine retinopathy (Rohrschneider et al., 2008). It is comparable to mfERG (inferior sensitivity but superior specificity) although this has not been widely performed or recommended for retinopathy screening due to limited availability (Jivrajka et al., 2013; Martinez-Costa et al., 2013; Iftikhar et al., 2019). Although it has a learning curve, and involves long examination time and limited by patient fatigue, this can be useful as an ancillary functional test in selected patients.

#### 5.1.3 Multifocal electroretinography (mfERG)

**FIGURE 7:**
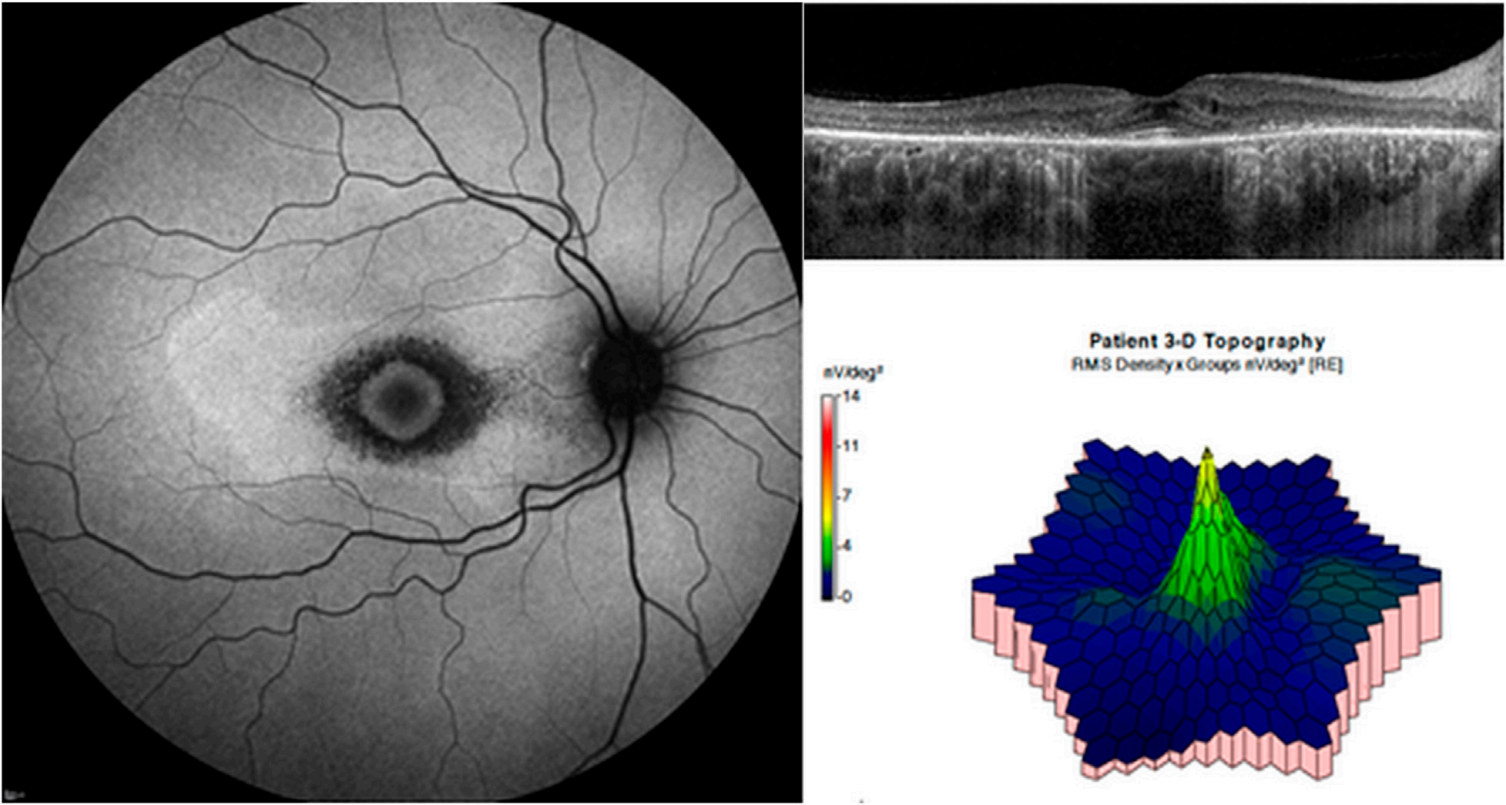
Multifocal electroretinography (mfERG) in a patient with parafoveal hydroxychloroquine retinopathy. Fundus autofluorescence (left) shows hypoautofluorescent ring on the parafoveal area. Optical coherence tomography (top right) reveals photoreceptor loss on the areas. On mfERG (bottom right), amplitude reduction is remarkable on the parafoveal area.

In contrast to full-field electroretinography, which has limited sensitivity for the detection of localized changes in the retina, mfERG can detect focal abnormalities in retinal sensitivity within the macula. Retinal responses are elicited by an array of black and white pixels on a monitor that alternate under computer control. This enables the response from specific regions within the macula to be calculated (Hood et al., 2012; Hoffmann et al., 2021). More specifically, a decreased amplitude of the response and prolonged implicit time can be observed in the areas with retinal damage (Table 4). For example, Maturi et al. showed a marked reduction in the mfERG amplitude of the central 16° field in a patient with bull’s eye hydroxychloroquine retinopathy (Maturi et al., 1999). In cases showing ring-shaped retinopathy, amplitude reduction on the parafoveal or pericentral ring-shaped area can be observed on mfERG (Figure 7), whereas advanced cases may present with generalized loss in mfERG response (Maturi et al., 2004). Lai et al. showed that serial mfERG assessment may detect the early stages of retinopathy (Lai et al., 2005).

Multifocal ERG is an objective, functional test that may be particularly useful to confirm the decreased function of the areas showing suspected hydroxychloroquine-related field loss (Lai et al., 2006). Previous reports showed that this technique has a high sensitivity, particularly in detecting retinopathy at an early stage, but with variable specificity (Tsang et al., 2015; Tsang et al., 2019). There has been a consensus that mfERG is more sensitive than FAF when the tests can be performed accurately and consistently (Tsang et al., 2015; Allahdina et al., 2019; Tsang et al., 2019). Meta-analysis of the diagnostic ability of mfERG showed that the pooled sensitivity and specificity for retinopathy detection were 90% and 52%, respectively, thus, requiring careful consideration for the likelihood of high false positive results (Tsang et al., 2015).

Several studies have been performed on mfERG for screening (Lyons and Severns, 2007; Tsang et al., 2019). Lyons et al. suggested that the ring ratio, defined as the ratios of the central ring amplitude (R1) to each of the peripheral ring amplitudes, could be used for the detection of early hydroxychloroquine retinopathy (Lyons and Severns, 2007). Many authors have noted that the foveal signal (R1) is more variable because of either fixation problems or macular changes, and by comparison, the outer ring is generally more stable (and now recommended by the International Society for Clinical Electrophysiology of Vision) (Hood et al., 2012; Hoffmann et al., 2021). Tsang and colleagues recently showed that Ring 2 P1 amplitude is the most sensitive mfERG parameter, with the area under the curve of 0.97 (Tsang et al., 2019).

Despite the potentials of mfERG for the early detection of hydroxychloroquine retinopathy, real-world screening in the US showed that mfERG was a less preferred screening modality (Costedoat-Chalumeau et al., 2015) as it is time-consuming, less accessible than retinal imaging, and requires excellent patient cooperation for reliable results. Moreover, the test is subject to variability and noise, depending on the technique, and it is challenging for inexperienced examiners to interpret reliably. Tracking the progression of hydroxychloroquine retinopathy using mfERG largely depends on patient cooperation and may also be affected by the test operator (for testing) or clinicians (for interpretation). Furthermore, test-retest variability is high even in normal subjects (Browning and Lee, 2014); hence, it seems impractical to diagnose retinopathy based on a mfERG test abnormality alone or to assess retinopathy progression using mfERG. The dependence on fixation should be carefully considered when interpreting the results of patients with poor fixation. Furthermore, SD-OCT or automated visual field testing are more widely available, limiting the need for mfERG as a routine screening test. A further limitation of mfERG is that it measures macular function; patients with the pericentral or peripheral retinal disease may exhibit false-negative results. Additionally, mfERG is not useful for advanced disease once the regional specificity is obvious from other tests, or the signals become universally low that regional specificity is absent.

#### 5.1.4 Full-field ERG

Full-field ERG (ffERG) is generally not considered a diagnostic tool for early screening or monitoring of hydroxychloroquine retinopathy; however, it may be of limited utility in more advanced cases as an indicator of severity and the breadth of damage. Ordinarily in “early” retinopathy before any bull’s eye change is visible in the fundus, ffERG remains normal as the extra macular damage is limited. However, sequential retinal thickness measurements (see below) can show diffuse tissue loss even in very early cases and in most cases with advanced bull’s eye change indicating clear loss of ERG signal ranging from mildly reduced amplitude and cone signal delay to essentially not recordable ERG responses mimicking dystrophies such as retinitis pigmentosa (Nair and Marmor, 2017).

### 5.2 Anatomic measures

#### 5.2.1 High-resolution (spectral-domain or swept-source) OCT

OCT imaging of the retina is the main screening modality for hydroxychloroquine retinopathy (Marmor et al., 2016). It is the most widely performed noninvasive test used for the diagnosis of retinal/macular diseases and provides high-resolution cross-sectional images of the retina. Structural assessment of the retina using OCT provides objective evidence of outer retinal damage caused by hydroxychloroquine toxicity. Owing to its limitations in the visualization of outer retinal changes, particularly early defects in the photoreceptors, time-domain OCT is no longer recommended (Marmor et al., 2016) and higher-resolution spectral-domain or swept-source OCT should be used.

Based on previous studies, the sensitivity of OCT in the detection of hydroxychloroquine retinopathy can range from 78.6% to 100% (Chen et al., 2010; Browning, 2014; Ahn et al., 2017a; Ahn et al., 2019b). These variable estimates of the sensitivity of OCT may relate to the subtle changes (outer nuclear layer thinning) observed in early disease in the absence of obvious qualitative alterations of outer retinal structures; such cases may be mistaken for normal examinations. To detect subtle outer nuclear layer thinning, retinal thickness measurements, either whole or specific layers (outer nuclear layer), may enhance the sensitivity of OCT in the context of hydroxychloroquine retinopathy.

The topographical distribution of retinal damage varies. Eyes with parafoveal involvement show outer retinal damage in the inner parafoveal field, whereas those with a pericentral pattern demonstrate outer retinal changes outside the parafoveal area, typically around the major vascular arcades. More peripheral retinal involvement may require particular attention regarding the scan length or area during OCT imaging, as the standard OCT scan might not cover the photoreceptor defects (Ahn et al., 2017a). This may lead to late or missed diagnosis in eyes with a pericentral pattern, which is more common in Asian patients (Melles and Marmor, 2015; Ahn et al., 2017a) but also noted in Caucasian populations. Accordingly, recent guidelines from the AAO recommended a wider-angle scan routinely for Asian patients (Marmor et al., 2016) and another study suggested multiple scans of at least 9-mm length (approximately 30°) covering the macular and extramacular areas, which may facilitate the early detection of hydroxychloroquine retinopathy in eyes with the pericentral disease (Ahn et al., 2017a).

If typical photoreceptor loss (Table 4) is identified in the perifoveal or paracentral areas on OCT images in both eyes of hydroxychloroquine users, this is strongly suggestive of the diagnosis. Collectively, in the era of modern retinal imaging, OCT plays an imperative role in hydroxychloroquine retinopathy screening as a key objective test.

#### 5.2.2 Fundus autofluorescence (FAF)

FAF is a noninvasive imaging modality that produces a density map of fluorophores in the RPE and can capture a wide area in a confocal plane within a single image (Yung et al., 2016). In general, hyper-autofluorescence indicates an increased accumulation of lipofuscin, which can be caused by alterations in lipofuscin metabolism (Kennedy et al., 1995). Hypo-autofluorescence may be due to reduced or absent lipofuscin, which may be caused by a loss of the RPE cells, such as in eyes with advanced hydroxychloroquine retinopathy (Yung et al., 2016). FAF has been considered as another useful objective, structural screening test for hydroxychloroquine retinopathy (Kellner et al., 2006).

In early hydroxychloroquine retinopathy, FAF imaging may demonstrate a parafoveal or pericentral hyper-autofluorescent region, which corresponds to the areas with photoreceptor damage, as shown in Figure 3. This hyper-autofluorescence may decrease over time if there is disease progression and RPE cell death; hypo-autofluorescence can be observed in the parafoveal or pericentral areas in eyes with severe disease. As the toxicity progresses, areas of hyper- or hypo-autofluorescence increase, and multiple hyper- or hypo-autofluorescent areas can coalesce (Ahn et al., 2020).

FAF changes may be absent or subtle in early disease, making FAF less sensitive than OCT particularly for early disease detection (Kellner et al., 2006; Marmor, 2013; Ahn et al., 2020). Multifocal ERG and standard automated perimetry are reported to be more sensitive than FAF imaging in detecting hydroxychloroquine retinopathy, particularly in early disease (Kellner et al., 2006; Marmor, 2013). Nevertheless, once abnormal, FAF provides a highly useful evaluation in hydroxychloroquine retinopathy as it indicates RPE involvement (as hypo-autofluorescence) and the topographic distribution of disease in one image, when compared to OCT. Severe hydroxychloroquine retinopathy can be easily determined by the presence of hypo-autofluorescence on FAF (see the right panel of Figure 3). In addition to the evaluation of disease severity, FAF provides information on the extent of the retinopathy, which can also be useful for the classification of earlier disease, between early (<180°) and moderate stages. Furthermore, the extent of retinal damage varies widely even in eyes with parafoveal retinopathy (Ahn et al., 2020) and the whole extent can be captured in a single image, particularly using wide-angle (200°) FAF imaging.

Wide-angle images, obtained using numerous devices, may be particularly useful in Asian populations (Ahn et al., 2020) as these populations typically show pericentral or more peripheral retinal toxicity. Wide-angle FAF may improve the detection and analysis of peripheral findings, providing a topographic view of retinal damage. This field of capture is currently not possible using OCT. Accordingly, several studies on the progression of retinopathy have used this imaging modality, as the progression of retinopathy may be most easily understood on FAF images. The progression to severe disease can be easily assessed by the conversion of hyper-autofluorescence to hypo-autofluorescence, which is also clinically relevant to the future progression of the disease (Pham and Marmor, 2019).

#### 5.2.3 Fundus photography and examination

In the current OCT era, fundus photography is no longer recommended as a standard objective screening test for hydroxychloroquine retinopathy. Fundus examination and photography can generally only detect severe retinopathy once RPE degeneration is advanced as the opportunity for stopping hydroxychloroquine therapy is missed. Retinal toxicity should be detected before observing any signs during the fundus examination (Marmor, 2016). However, fundus photography or examination is one of the routine, low-cost tests performed in ophthalmology clinics. Therefore, the AAO guideline (2016) designated color fundus photography as a baseline (recommended within 1 year of hydroxychloroquine use) examination performed to exclude pre-existing retinal or macular diseases. However, color photography does not form part of the RCOphth monitoring guidelines (2020) (Yusuf et al., 2021b).

### 5.3 Measuring hydroxychloroquine blood levels for prediction of retinopathy

Studies have shown that higher blood levels of hydroxychloroquine may predict the development of hydroxychloroquine retinopathy. (Petri et al., 2020a;b). Interestingly, however, there is no strong correlation between a weight-based dose of hydroxychloroquine and hydroxychloroquine blood level. This may relate to variability between individuals in compliance, drug metabolism and excretion. Hydroxchloroquine levels, measured in the blood, may theoretically enable more precise dosing with regards to efficacy and safety. However, measurement of blood levels of hydroxychloroquine is not widely available, and may be unhelpful at certain stages due to a long time to reach steady state in the retina. Furthermore, natural history studies of hydroxychloroquine retinopathy do not frequently include measurement of blood levels, and consequently, the relationship is not as clearly defined as classic risk factors (i.e., daily dose, duration of use).

Regular monitoring of hydroxychloroquine plasma levels can particularly help to identify patients who are at a higher risk of developing retinopathy due to renal impairment, where the risk of retinopathy is known to be higher. However, there is no clear guidance on the utility of blood hydroxychloroquine monitoring in any current clinical guideline on screening for retinopathy.

### 5.4 Screening recommendations

Ophthalmologists and rheumatologists should be aware of the current best practice recommendations established by ophthalmological and rheumatologic societies for the prevention (safe dosing) and early detection of retinopathy (screening; monitoring is the preferred term in the U.K.). These recommendations were formulated using different methodologies. However, they aim to identify a standard that reduces variations in practice. The organizations that have published recommendations in the context of hydroxychloroquine retinopathy include national societies in the fields of ophthalmology (AAO and RCOphth), rheumatology (American College of Rheumatology [ACR] and British Society of Rheumatology [BSR]), and dermatology (American Academy of Dermatology [AAD] and Rheumatologic Dermatology Society).

**FIGURE 8:**
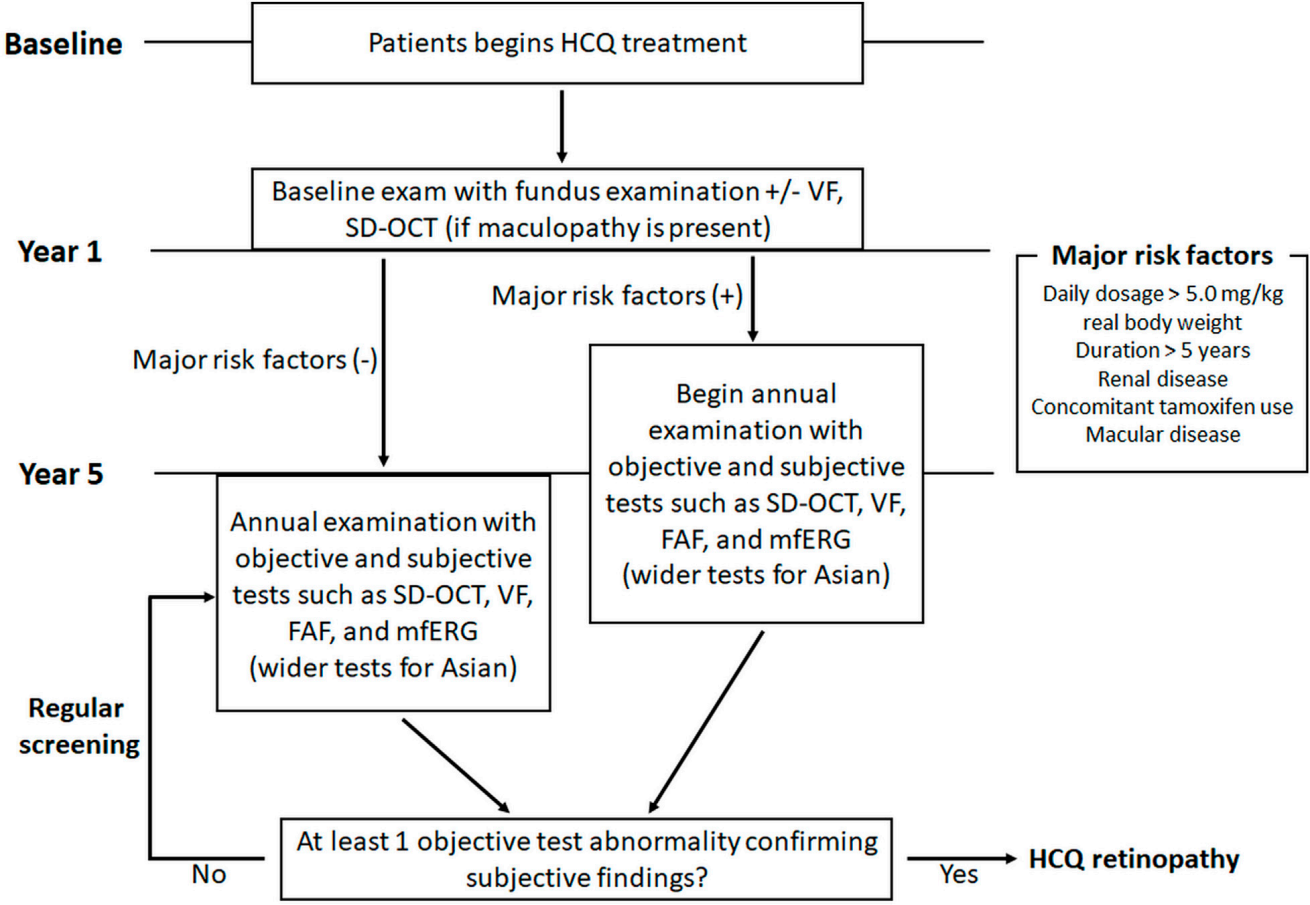
Screening algorithms of hydroxychloroquine retinopathy as reported by the American Academy of Ophthalmology in 2016. FAF = fundus autofluorescence; mfERG = multifocal ERG; SD-OCT = spectral-domain optical coherence tomography; VF = automated visual field.

The AAO recommendations for screening chloroquine and hydroxychloroquine retinopathy were first published in 2002. With advances in retinal imaging and knowledge of the disease, the recommendations were revised in 2011 and 2016. The key changes to the previous recommendations were the establishment of a major risk factor based on the ratio of daily dose to real body weight (5 mg/kg) and adaptation of screening examinations for Asian patients. A flowchart indicating the sequence of diagnostic tests recommended by the most recent AAO guidelines is shown in Figure 8.

In 2016, the RCOphth in the UK convened a guideline development group whose primary purpose was to undertake a systematic evaluation of the literature and determine whether monitoring for users of hydroxychloroquine and chloroquine was justified. Previous RCOphth committees concluded that there was insufficient evidence at the time of evaluation to support systematic monitoring for retinopathy across the National Health Service in 1997 (Silman and Shipley, 1997) and 2009 (Bourke et al., 2009). The evidence was systematically reviewed in collaboration with the Cochrane Eyes and Vision Group for all studies published between 2000 and 2017, screened by the guideline development group, and graded according to the Scottish Intercollegiate Grading Network framework into the type of evidence, and then formed into recommendations based on the strength of evidence (Yusuf et al., 2018). The committee included rheumatologists, dermatologists, optometrists, and primary care physicians (Lotery and Yusuf, 2018; Yusuf et al., 2019). The recommendations were refined in 2020 following the publication of two large audits of monitoring services performed as per the 2018 guidelines. Of note, these recommendations deviate from those of the AAO. Of particular interest is the timing and techniques of examination recommended for baseline examinations, if any, and during screening episodes.

#### 5.4.1 Timing

The baseline examination aims to identify pre-existing macular disorders and other patient-related or ocular factors that might limit the ability to screen an individual at risk. Screening examinations are generally undertaken at annual intervals and are performed according to pre-designated testing protocols. However, the timing of the first screening episode may vary depending on the presence of any additional risk factors in a given individual.

Baseline examination is recommended within a year of hydroxychloroquine treatment initiation according to the 2011/2016 AAO guidelines (Figure 8). However, the RCOphth no longer recommends baseline examination in hydroxychloroquine initiators in the UK (2020 recommendations (Yusuf et al., 2021b)). Two large audits of screening practices undertaken in accordance with the RCOphth recommendations from 2018 (Yusuf et al., 2018) found that baseline testing was most often non-contributory: only 26 of 1,127 individuals (pooled frequency of 2.3%) were found to have ocular comorbidities on baseline testing, suggesting they were unsuitable for monitoring (Gobbett et al., 2021; Marshall et al., 2021). Consequently, baseline testing was not considered cost-effective and was no longer recommended as part of the RCOphth recommendations 2020 (Yusuf et al., 2021a; Yusuf et al., 2021b). However, retinal imaging at treatment initiation can be helpful in the later interpretation of retinal abnormalities. Therefore, the role and cost-effectiveness of baseline examination needs further discussion.

The 2011/2016 AAO guidelines recommended annual monitoring in patients with 5 years of hydroxychloroquine exposure but without major risk factors (Figure 8). Nevertheless, if patients have one or more major risk factors (daily dose >5 mg/kg real body weight), annual or more frequent screening should be initiated sooner. Compared to the previous version (2011), the 2016 revision modified the major risk factors. For example, the criteria for cumulative dose were omitted, and the daily dosage was based on real body weight. Age and liver dysfunction are no longer major risk factors, whereas kidney disease (stage 3 or worse) remains a risk factor. Furthermore, the AAO guidelines emphasize regular review of the major risk factors, as they are not invariable over the long term (a change in body weight or the development of renal impairment may modify risk). Accordingly, patients should be asked whether there have been changes in body weight, kidney disease, and tamoxifen use at every visit.

**FIGURE 9:**
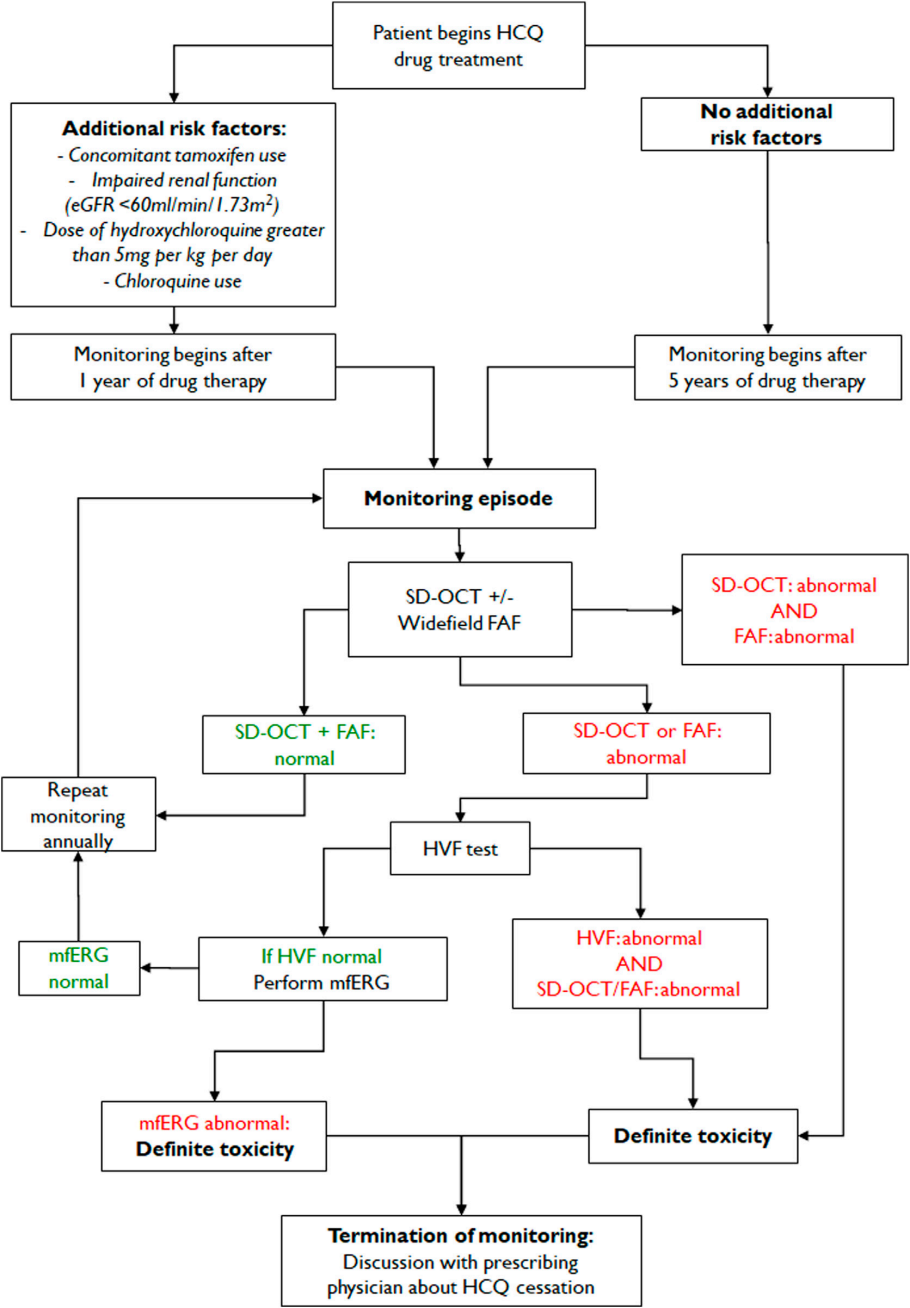
Monitoring algorithm of hydroxychloroquine retinopathy recommended by the Royal College of Ophthalmologists (proposed 2020 revision). Note the absence of baseline testing and the retinal imaging-based monitoring algorithm. HVF—Humphrey visual field; SD-OCT—Spectral domain optical coherence tomography imaging; mfERG—multifocal electroretinography; FAF—fundus autofluorescence imaging.

In the UK, the joint monitoring recommendations published by the RCOphth guideline development group aligned with the AAO guidelines regarding the timing of screening (Lotery and Yusuf, 2018; Yusuf et al., 2018; Yusuf et al., 2019). However, the recently updated RCOphth guidelines (2020) differ from those published in 2018 and the AAO guidelines. (Figure 9). In the updated guidelines, annual monitoring should be initiated after 5 years of therapy and performed annually thereafter, while on therapy. However, if patients have additional risk factors such as a high daily dose (>5 mg/kg/day), concomitant tamoxifen use, and impaired renal function, annual monitoring is recommended to initiate after 1 year of hydroxychloroquine therapy. As baseline examination is no longer required, no distinction between baseline and annual screening in this guideline exists, and monitoring can be identically performed if structural tests are normal.

#### 5.4.2 Techniques of examination

The tests used to screen for hydroxychloroquine retinopathy aim, in combination, to detect the earliest pre-symptomatic signs of retinal toxicity. The screening protocols must consider the relative sensitivity and specificity of each test and for the tests in combination to detect toxicity. Additionally, access and availability of the tests, acceptability to patients, and impact on healthcare resources (particularly in resource-poor settings) must all be considered in any recommendation. The AAO guidelines stipulate that both subjective and objective evidence of toxicity must be present to confirm toxicity; this generally involves both structural and functional tests.

In the most recent AAO guideline (2016), SD-OCT and automated visual fields were recommended for the baseline examination, particularly if a focal macular lesion or glaucoma were present on dilated ocular examination. For screening examinations, the AAO guidelines require at least one objective test to confirm the subjective test abnormalities. The standard tests recommended by the AAO guidelines include SD-OCT, automated visual fields, FAF, and mfERG.

Although protocols used for the examinations are not specified in the 2016 AAO recommendations, they emphasize the differential use of protocols or wide-angle screening scans for Asian eyes. For instance, the 2011 guideline recommended a 10-2 protocol for automated visual field tests in all patients receiving hydroxychloroquine retinopathy screening; however, the 2016 guideline suggested wider test patterns (24-2 or 30-2) for visual field test and wider-angle images of SD-OCT and FAF for Asian patients (Marmor et al., 2016).

However, in the most recent RCOphth guidelines (2020) restructured the order of tests undertaken during screening examinations based on published data (Figure 9). New data suggest that visual field testing is a significant source of inefficiency within the screening service and a barrier to screening coverage across the healthcare system (Yusuf et al., 2021b). Specifically, Marshall et al. and Gobett et al. found that 33.1% and 17% of visual fields, respectively, were unreliable (Gobbett et al., 2021; Marshall et al., 2021). Moreover, visual field testing consumes significant resources with regard to time, staff, and the capacity for screening services. Therefore, the RCOphth recommendations (2020) were revised in response to these new data (Yusuf et al., 2021a) and currently recommend spectral-domain OCT and FAF (widefield if available) for all individuals at monitoring visits. Visual field testing is recommended only as a second-line test, performed only if abnormalities consistent with hydroxychloroquine retinopathy are present on either retinal imaging test. The visual field test protocol can then be targeted to the anatomical location of the defect (24-2 or 30-2 for pericentral defects). For individuals with one abnormal structural test result and a full visual field, mfERG may be considered.

In the RCOphth guideline, definite retinal toxicity is defined as two abnormal test results consistent with hydroxychloroquine retinopathy to limit the potential harm of inappropriate treatment cessation on a single test result. There is no specification regarding the nature of the tests (subjective or objective); congruous abnormalities on SD-OCT and FAF consistent with toxicity would be sufficient for the diagnosis of “definite toxicity.” The rearrangement of the test protocol does not affect the diagnostic yield, but greatly reduces the national cost of screening (Yusuf et al., 2021b).

#### 5.4.3 Formal recommendations by rheumatologic organizations

Guidelines of rheumatologic societies provide recommendations on the safe dosing of hydroxychloroquine to reduce the risk of retinopathy. The ACR stated that patients treated with hydroxychloroquine should undergo baseline and periodic eye examinations for the early detection of retinal toxicity before patients recognized visual loss (American College of Rheumatology, 2016). However, the timing and extent of surveillance monitoring have not been made explicit. For instance, according to the recommendation, all individuals using the drug should undergo a complete baseline ophthalmologic examination within the first year of the treatment, including retinal examination with pupil dilation, visual fields by an automated central visual field test, and, if available, objective tests such as mfERG, SD-OCT, and FAF. However, the interval of monitoring had not been specified; instead, the timing was suggested to be based on the individual risk of retinopathy. Interestingly, the guidelines recommend that the rheumatologist, ophthalmologist, and patient must reach a collective decision to stop the drug or cautiously continue the therapy with close monitoring of the systemic drug load and the risk of further vision loss. The BSR recommends an annual eye assessment, ideally including SD-OCT, if hydroxychloroquine is to be used for more than 5 years (Ledingham et al., 2017). However, the BSR recommendations predate the most recent RCOphth recommendations, which no longer support baseline testing.

The EULAR recently updated recommendations for the management of SLE (Fanouriakis et al., 2019). Based on the up-to-date evidence, the recommendation suggested hydroxychloroquine should be used in all patients with SLE. However, due to the risk of retinal toxicity, more sensitive screening techniques should be used for the screening of retinopathy, and the daily hydroxychloroquine dose should not exceed 5 mg/kg real body weight. This recommendation on safe dose is in accordance with the study by Melles et al., who used actual body weight and the same threshold for the criteria (Melles and Marmor, 2014).

#### 5.3.4 Dermatology organization recommendations

Expert opinions or organizational recommendations have been provided recommendations on the use of hydroxychloroquine in dermatologic practices (Fernandez, 2017; Yusuf et al., 2021c; Rosenbaum et al., 2021). Dermatology organizations also collaborated recommendations for the safe dosing of hydroxychloroquine and screening practices of retinal toxicity with rheumatological and ophthalmological societies (Ding et al., 2016). The British Association of Dermatologists endorsed the recent RCOphth guidelines and American Academy of Dermatology and Rheumatologic Dermatology Society participated in the joint statement on hydroxychloroquine use with respect to retinal toxicity (Rosenbaum et al., 2021). This recent joint statement by ACR, American Academy of Dermatology, Rheumatologic Dermatology Society, and AAO highlighted effective communication among prescribing physicians, patients, and eye care providers for optimizing the utility of hydroxychloroquine while ensuring the safety of the drug. Regarding the safe dosing, daily dosage should not exceed 5 mg/kg actual body weight. In the statement, baseline testing is advised when starting hydroxychloroquine to rule out confounding disease while annual screening with sensitive modalities such as OCT and automated visual fields should begin no more than 5 years later. Finally, the guideline suggested that prescribing physicians should not stop this valuable drug for uncertain findings (Rosenbaum et al., 2021).

## 6 Management and treatment of hydroxychloroquine retinopathy

### 6.1 Clinical course after drug cessation

Using modern multimodal retinal imaging techniques, Mititelu et al. documented the progression of hydroxychloroquine retinopathy after drug cessation (Mititelu et al., 2013). Outer retinal atrophy, seen as photoreceptor loss and thinning of the RPE complex on OCT and new or enlarged hypo-autofluorescence on FAF, was noted following drug cessation in eyes with advanced retinopathy. However, five of seven patients experienced outer retinal regeneration, with associated functional visual improvement in two and FAF stability in three patients (Mititelu et al., 2013). This regeneration, partial recovery of photoreceptor defects on OCT, has also been noted in subsequent studies (Pham and Marmor, 2019; Ahn et al., 2021). These observations indicate that either recovery or progression of hydroxychloroquine retinopathy may occur after drug cessation, which appears to depend on the disease stage and on the sensitivities of modalities used for retinal imaging.

As the number of patients with hydroxychloroquine retinopathy increase and long-term data on the natural history of retinopathy accumulate, longitudinal changes in retinopathy in parafoveal and pericentral patterns are becoming clearer. Pham et al. showed longitudinal changes in retinopathy, up to 20 years after drug cessation, in eyes with parafoveal retinopathy, which showed continuous progression of severe retinopathy (Pham and Marmor, 2019). In contrast, eyes with early or moderate retinopathy demonstrated a stable course over a long period after drug cessation. Several reports have shown comparable results regarding the progression of retinopathy, with progression more likely in eyes with severe retinopathy (Allahdina et al., 2019). However, a recent report on pericentral retinopathy showed a different natural history to parafoveal disease. Moderate pericentral retinopathy generally progresses to severe retinopathy, although it can be stable in selected cases (Ahn et al., 2021). It is uncertain why the natural history of parafoveal and pericentral retinopathy appears to differ in moderate eyes.

Moreover, it is unclear what determines the post-cessation behavior of hydroxychloroquine retinopathy. Mititelu et al. suggested the preservation of the external limiting membrane on OCT as a positive prognostic factor for the regeneration of the photoreceptor layers following drug cessation (Mititelu et al., 2013). However, this has not been specifically verified independently of other morphological alterations on OCT imaging in confirmed cases of retinopathy.

#### 6.1.1 Is there ever recovery?

**FIGURE 10:**
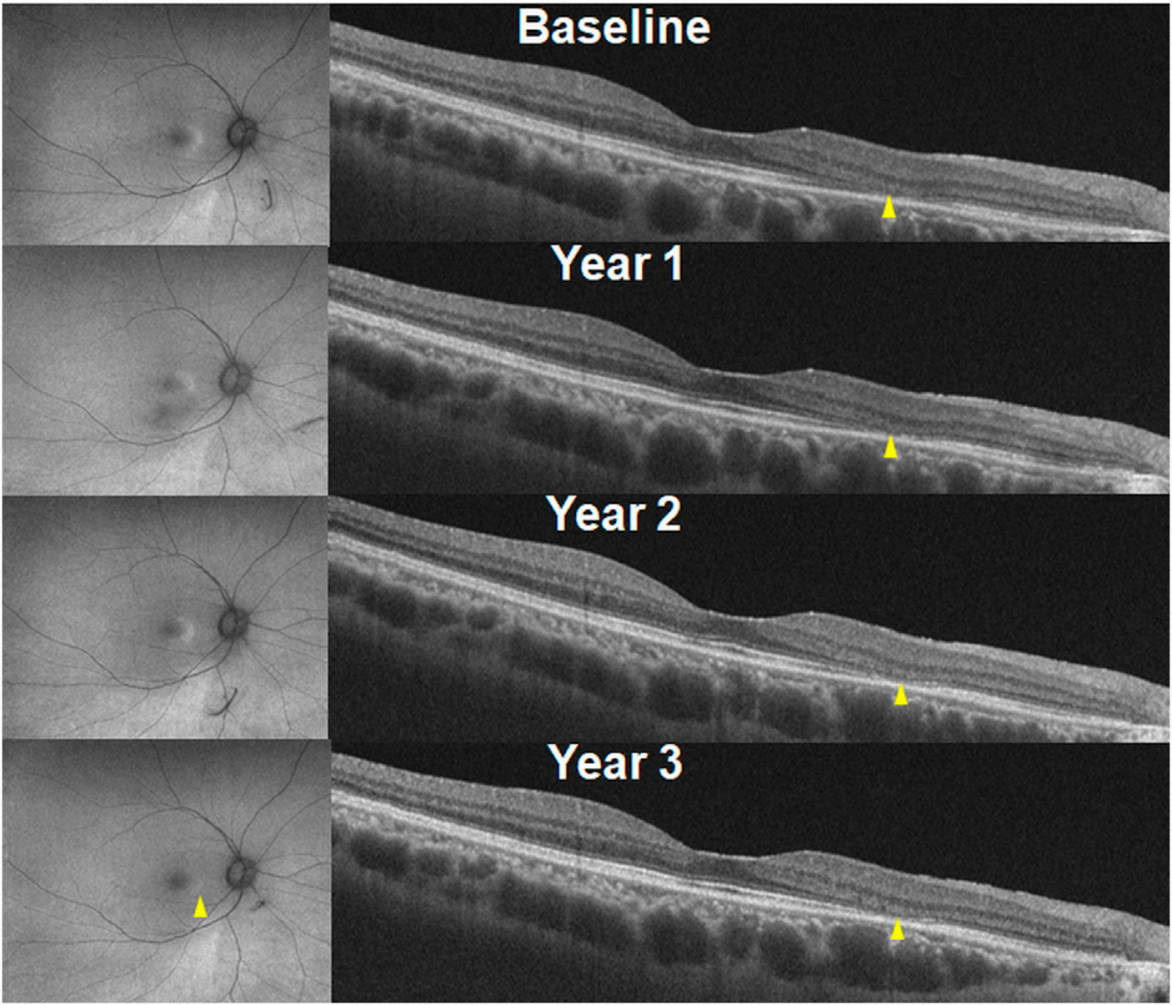
Recovery of hydroxychloroquine retinopathy in an eye with early retinopathy following drug cessation. Optical coherence tomography (right) images indicate a decrease in the length of photoreceptor defects (indicated by arrowheads). This partial recovery is also remarkable in fundus autofluorescence (left) in the parafovea (arrowhead). (Reprinted with permission from Ahn et al. Ophthalmology. 2021; 128:889-898).

It is generally believed that hydroxychloroquine retinopathy is irreversible. However, using modern retinal imaging techniques, several reports have shown objective evidence of recovery after drug cessation (Mititelu et al., 2013; Pham and Marmor, 2019). For instance, five of seven patients with outer retinal regeneration had perimetric improvement following drug cessation (Mititelu et al., 2013). Pham et al. also showed sequential changes of partial structural improvement in early cases of hydroxychloroquine retinopathy (Pham and Marmor, 2019). A recent report on the long-term progression of pericentral retinopathy also illustrated the recovery process from a mild case (Figure 10) (Ahn et al., 2021). However, functional recovery was not evident despite structural recovery on OCT (Ahn et al., 2021). Based on evidence from OCT imaging of objective structural improvements in multiple studies, early retinopathy can be now considered as partly reversible. However, a direct correlation with microperimetry in such cases would be of great interest to determine whether the structural recovery is accompanied by functional improvement. Furthermore, the reversibility of photoreceptor damage should be validated from long-term data to determine whether such recovery process is sustained or complete. Functional recovery on multifocal ERG, defined as an improvement in the R1/R2 ratio, was identified in early hydroxychloroquine retinopathy (Allahdina et al., 2019), but not in those with more advanced disease. Structurally, continuous progression in advanced diseases was noted in both parafoveal and pericentral retinopathy (Pham and Marmor, 2019; Ahn et al., 2021).

Collectively, these data suggest that individuals with early disease may exhibit structural and/or functional signs of objective recovery. However, the threshold for the recovery or predictors of photoreceptor recovery in hydroxychloroquine retinopathy is still unknown and requires further investigation.

### 6.2 Dose lowering vs cessation

There is no proven therapy that prevents hydroxychloroquine retinopathy or ameliorates the clinical course of the disease. Drug cessation is the only management strategy that has some evidence of reducing the risk of disease progression. The natural history data summarized above support the role of screening in the detection of the most subtle manifestations of the disease to discontinue treatment at a stage at which retinopathy will not progress. The threshold of disease that can be detected using screening requires further consideration based on these data.

Hydroxychloroquine has been used in RA to reduce the dose of other medications with more severe adverse effects or to justify the use of biologic agents that require treatment failure with two or more agents before consideration. However, hydroxychloroquine has been shown to confer a survival benefit in patients with SLE, and its omission therefore requires clear justification. It remains to be determined whether the same disease-modifying effect is observed at safe (<5 mg/kg/day) or at lower doses. Therefore, the decision on dose lowering or drug cessation should be made based on the therapeutic indication and clinical effectiveness of the lowered dose in the target disease. Generally, it may be reasonably considered by physicians and their patients that hydroxychloroquine is a safer disease-modifying agent than most others in SLE and RA, and the risk of retinal toxicity is acceptable and minimized by screening procedures even at doses in the toxic range. Alternatively, a dose <5 mg/kg may be recommended to reduce the risk of retinopathy at the expense of some therapeutic benefit. These remain key unanswered questions.

It appears compelling that alternative medications should be considered for those with retinal toxicity; however, dose reduction may be an option for those with mild to moderate disease, particularly for individuals who derive significant clinical benefit from the drug. For individuals with advanced disease characterized by a loss of RPE, drug cessation would probably be recommended. Most importantly, all individuals with proven toxicity should be carefully counseled based on the risk of loss of vision, impact on systemic disease control, and availability of other treatment options involving clear communication between the ophthalmologists and prescribing physicians.

#### 6.2.1 When to stop: Medical need *versus* ophthalmic concern for progression or loss of central vision

The decision to stop the medication will be based on the balance between the nature of the clinical indication, the clinical efficacy for the treatment indication, severity of retinopathy and the risk of progression, which requires input from ophthalmologists. Since control of the underlying rheumatologic/inflammatory disease can be adversely affected by treatment cessation, a clear understanding of the severity of retinopathy is vital for prescribers to make management decisions.

Hydroxychloroquine may be discontinued at the time of diagnosis by prescribing physicians. However, the diagnosis of retinopathy should be made or confirmed by an ophthalmologist based on sufficient evidence to avoid inappropriate drug cessation. To obtain sufficient evidence on retinal toxicity, further testing is required to verify the isolated findings in a diagnostic test before toxicity is confirmed. Considering this, the most recent recommendations from the AAO suggested “at least one objective test abnormality confirming the subjective abnormality” for the diagnosis (Marmor et al., 2016). Furthermore, the RCOphth recommends two abnormal and congruent test results consistent with hydroxychloroquine retinopathy to define “definite toxicity” and recommends treatment cessation, depending on the individual case (Yusuf et al., 2021b).

An alternative approach is that hydroxychloroquine can be discontinued when there is a risk of vision-threatening retinopathy, although further research is required to identify this threshold. Most patients with subtle hydroxychloroquine retinopathy are asymptomatic unless retinopathy involves central vision. The progression of hydroxychloroquine retinopathy is known to be slow; therefore, patients with the subtle disease and preserved visual acuity and central visual field may continue the drug if the medical need outweighs the risk of progression of the retinopathy. In this context, patients should be counseled regarding the risk of progression of the retinopathy *versus* flare of the underlying disease. Moreover, such patients require continued monitoring of their retinopathy, perhaps more frequently than annually. Dose adjustment, in addition to regular monitoring, can be another option for appropriate management in cases with particular medical needs. However, the risk of progression to the involvement of central vision is significant for eyes with severe retinopathy (Mititelu et al., 2013; Pham and Marmor, 2019; Ahn et al., 2021). Prescribing physicians should be made aware of such risks in advance of discussions with individual patients on treatment cessation.

### 6.3 Other therapeutic options

There are no published data suggesting a protective or harmful effect of any particular diet or vitamin supplementation on the incidence or progression of hydroxychloroquine retinopathy.

One unique case report showed more severe retinopathy in the phakic eye than in the aphakic eye, suggesting a possible modifying effect of light exposure in the development of retinopathy (Mack et al., 2018). However, there are no supporting data suggesting an increased prevalence of toxicity in a population with greater sun exposure. Currently, there is insufficient evidence to support light avoidance in hydroxychloroquine users.

Currently, there is no proven therapy that has been shown to modify the clinical course of hydroxychloroquine retinopathy. Pharmacological approaches that can achieve photoreceptor recovery or provide neuroprotection against photoreceptor degeneration might also be effective in minimizing the functional loss caused by retinopathy. Several experimental studies have suggested candidate molecules for the treatment of hydroxychloroquine retinopathy. For example, the molecules normalizing lysosomal pH by increasing cAMP or free zinc levels in RPE cells (zinc ionophore clioquinol (Seo et al., 2015)) or those reducing autophagy (e.g., n-acetyl cysteine) could be a promising therapeutic regimen for chloroquine or hydroxychloroquine toxicity (Koh et al., 2019). For the discovery of chemicals that prevent the development or progression of retinopathy or even recover the damaged photoreceptors, the pathogenic pathways for the retinopathy should be elucidated from appropriate *in vitro* (cellular) or *in vivo* (animal) models.

## 7 Conclusion

Hydroxychloroquine is increasingly used for the management of various rheumatological and dermatological disorders, and the number of long-term hydroxychloroquine users is increasing. With the use of modern retinal imaging techniques, the prevalence of hydroxychloroquine retinopathy is higher than previously estimated. Currently, four screening tests, SD-OCT, FAF, mfERG, and automated visual field examination, are recommended for hydroxychloroquine retinopathy, while OCT and automated visual fields are the established, widely available, and accessible tests for disease detection. Nationally agreed consensus statements or guidelines, which have been established by several ophthalmology and rheumatology societies, provide safe dose recommendations (the daily dose should be less than 5 mg/kg of the real body weight), and guidelines on the timing and techniques of regular monitoring based on the underlying major risk factors such as daily dose per kilogram of body weight, duration of hydroxychloroquine use, coexistent kidney/macular diseases, and use of tamoxifen. These guidelines should be more widely disseminated among prescribing physicians and ophthalmologists, implemented in real-world clinical practice, and audited to ensure compliance and quality control of procedures. Although no treatment other than drug cessation has been proven effective for the management of hydroxychloroquine retinopathy, the decision on drug termination should be carefully taken through communication with prescribing physicians. Thus, effective communication and cooperation are required between prescribing physicians and ophthalmologists to minimize the risk of retinal toxicity and its progression.
